# Fragment screening and structural analyses highlight the ATP-assisted ligand binding for inhibitor discovery against type 1 methionyl-tRNA synthetase

**DOI:** 10.1093/nar/gkac285

**Published:** 2022-04-26

**Authors:** Jia Yi, Zhengjun Cai, Haipeng Qiu, Feihu Lu, Zhiteng Luo, Bingyi Chen, Qiong Gu, Jun Xu, Huihao Zhou

**Affiliations:** Guangdong Provincial Key Laboratory of Chiral Molecule and Drug Discovery, School of Pharmaceutical Sciences, Sun Yat-sen University, Guangzhou 510006, China; Research Center for Drug Discovery, School of Pharmaceutical Sciences, Sun Yat-sen University, Guangzhou 510006, China; Guangdong Provincial Key Laboratory of Chiral Molecule and Drug Discovery, School of Pharmaceutical Sciences, Sun Yat-sen University, Guangzhou 510006, China; Research Center for Drug Discovery, School of Pharmaceutical Sciences, Sun Yat-sen University, Guangzhou 510006, China; Guangdong Provincial Key Laboratory of Chiral Molecule and Drug Discovery, School of Pharmaceutical Sciences, Sun Yat-sen University, Guangzhou 510006, China; Research Center for Drug Discovery, School of Pharmaceutical Sciences, Sun Yat-sen University, Guangzhou 510006, China; Guangdong Provincial Key Laboratory of Chiral Molecule and Drug Discovery, School of Pharmaceutical Sciences, Sun Yat-sen University, Guangzhou 510006, China; Research Center for Drug Discovery, School of Pharmaceutical Sciences, Sun Yat-sen University, Guangzhou 510006, China; Guangdong Provincial Key Laboratory of Chiral Molecule and Drug Discovery, School of Pharmaceutical Sciences, Sun Yat-sen University, Guangzhou 510006, China; Research Center for Drug Discovery, School of Pharmaceutical Sciences, Sun Yat-sen University, Guangzhou 510006, China; Guangdong Provincial Key Laboratory of Chiral Molecule and Drug Discovery, School of Pharmaceutical Sciences, Sun Yat-sen University, Guangzhou 510006, China; Research Center for Drug Discovery, School of Pharmaceutical Sciences, Sun Yat-sen University, Guangzhou 510006, China; Research Center for Drug Discovery, School of Pharmaceutical Sciences, Sun Yat-sen University, Guangzhou 510006, China; Research Center for Drug Discovery, School of Pharmaceutical Sciences, Sun Yat-sen University, Guangzhou 510006, China; Guangdong Provincial Key Laboratory of Chiral Molecule and Drug Discovery, School of Pharmaceutical Sciences, Sun Yat-sen University, Guangzhou 510006, China; Research Center for Drug Discovery, School of Pharmaceutical Sciences, Sun Yat-sen University, Guangzhou 510006, China

## Abstract

Methionyl-tRNA synthetase (MetRS) charges tRNA^Met^ with l-methionine (L-Met) to decode the ATG codon for protein translation, making it indispensable for all cellular lives. Many gram-positive bacteria use a type 1 MetRS (MetRS1), which is considered a promising antimicrobial drug target due to its low sequence identity with human cytosolic MetRS (*Hc*MetRS, which belongs to MetRS2). Here, we report crystal structures of a representative MetRS1 from *Staphylococcus aureus* (*Sa*MetRS) in its apo and substrate-binding forms. The connecting peptide (CP) domain of *Sa*MetRS differs from *Hc*MetRS in structural organization and dynamic movement. We screened 1049 chemical fragments against *Sa*MetRS preincubated with or without substrate ATP, and ten hits were identified. Four cocrystal structures revealed that the fragments bound to either the L-Met binding site or an auxiliary pocket near the tRNA CCA end binding site of *Sa*MetRS. Interestingly, fragment binding was enhanced by ATP in most cases, suggesting a potential ATP-assisted ligand binding mechanism in MetRS1. Moreover, co-binding with ATP was also observed in our cocrystal structure of *Sa*MetRS with a class of newly reported inhibitors that simultaneously occupied the auxiliary pocket, tRNA site and L-Met site. Our findings will inspire the development of new MetRS1 inhibitors for fighting microbial infections.

## INTRODUCTION

The imprudent use of antibiotics has boosted the emergence of multidrug-resistant superbugs, such as methicillin-resistant *Staphylococcus aureus* (MRSA), which are involved in devastating infections (1). A recent report noted that MRSA was responsible for more than 100 000 deaths, which were specifically attributable to antimicrobial resistance (AMR) in 2019 (2). The threat from AMR partly reflects the limited access to effective antibiotics, and there is an urgent need for new antibiotics to combat drug-resistant bacterial pathogens (3).

The translation of genetic codes into protein sequences requires the specific ligation of amino acids to their corresponding tRNAs, which is catalyzed by aminoacyl-tRNA synthetases (AARSs) (4,5). Methionyl-tRNA synthetase (MetRS) is an AARS member that catalyzes the esterification of L-Met to both initiator and elongator methionine tRNAs (tRNA^fMet^ and tRNA^Met^) (6). MetRS is a multidomain protein with a minimal catalytic core that consists of an aminoacylation domain (AD), a connecting peptide (CP) domain, a stem contact fold (SCF) and a helical anticodon-binding domain (ABD) (7). In addition, N- and/or C- terminal polypeptide extensions are frequently appended to MetRS from different organisms (8–10). Sequence and structural analyses suggest that MetRS can be divided into two types, MetRS1 and MetRS2, which are characterized mainly by their knuckle structure in the CP domain (11). Compared to MetRS1, the CP domain of MetRS2 has an additional insertion sequence of ∼20 amino acids that form the second knuckle (12). MetRS1 proteins typically exist in gram-positive bacteria, protozoan parasites and mitochondria, while MetRS2 proteins are mostly found in eukaryotes, archaea and gram-negative bacteria (13).

Owing to the irreplaceable roles of AARSs in protein synthesis, they have long been studied as attractive drug targets for fighting infectious diseases (14–16). Prominent examples include the AARS inhibitors mupirocin and tavaborole, which are used in clinics for treating bacterial and fungal infections, respectively (17,18). The sequence and structural diversity of MetRS from different organisms make it an ideal antimicrobial drug target. GlaxoSmithKline (GSK) reported a series of diaryldiamine-based compounds as potent inhibitors of *S. aureus* MetRS (*Sa*MetRS) through high-throughput screening (HTS) (19,20). Thereafter, many diaryldiamine derivatives have been developed to selectively target MetRS1 from *S. aureus* (*Sa*MetRS), *Trypanosoma brucei* (*Tb*MetRS) and *Clostridioides difficile* (*Cd*MetRS) (21–24), including compounds REP8839 and CRS3123, which have entered clinical trials (25,26).

All three aminoacylation substrates (amino acids, ATP and tRNA) have their own binding sites on AARSs. Most AARS inhibitors target amino acid and/or ATP binding sites, including mupirocin (27,28). To accelerate AARS-based drug discovery, inhibitors with new chemical scaffolds and novel inhibitory mechanisms are desired. Interestingly, crystallographic studies revealed that the diaryldiamine series compounds inhibit MetRS1 by occupying an enlarged amino acid binding site and a novel auxiliary pocket near the tRNA binding site in the active site cavity (29,30). Moreover, REP8839 was reported to exhibit enhanced inhibitory activity against MetRS1 at higher ATP concentrations (25), implying the activity is superior to that of traditional ATP competitive inhibitors in bacterial cells in which ATP typically reaches millimolar concentrations. Thus, these previous studies have suggested that there are great opportunities for discovering new mechanistic MetRS1-specific inhibitors, and the significance of ATP assistance as well as the auxiliary pocket for MetRS1 inhibitor development deserves careful investigation.

Here, we solved the structures of *Sa*MetRS in the apo and substrate-binding states, which revealed ligand-induced conformational changes that were different from those of human cytosolic MetRS (*Hc*MetRS). We then screened a library of 1049 chemical fragments against *Sa*MetRS that was saturated with or without ATP by using a fluorescence-based thermal shift assay (TSA) and identified ten fragment hits. Seven of these fragments bound to *Sa*MetRS only in the presence of ATP. The cocrystal structures of *Sa*MetRS with four fragments were successfully solved, which revealed that fragments M3-88 and M2-80 occupy the auxiliary pocket of *Sa*MetRS and M3-146 and B54 occupy the amino acid site. Isothermal titration calorimetry (ITC) assays confirmed that ATP favored the binding of M3-88 to the auxiliary pocket. Finally, cocrystal structures of a class of novel inhibitors that were reported recently were solved, and their binding modes further supported the importance of the auxiliary pocket as well as ATP assistance in developing MetRS1 inhibitors.

## MATERIALS AND METHODS

### Cloning, protein expression and purification

C-terminal domain truncated *Sa*MetRS (*Sa*MetRS-dC) (residues 1–520) was used in the protein crystallization assay. The gene encoding this truncated protein was amplified by PCR from the genomic DNA of *Staphylococcus aureus* and incorporated into the pET15b plasmid with an N-terminal His_6_-tag. *Sa*MetRS was expressed in *E. coli* BL21 (DE3) cells. Cells were grown in Luria-Bertani (LB) medium supplemented with 100 mg/l ampicillin at 37°C until the OD_600_ reached ∼0.6, and then overexpression of the target protein was induced by adding 0.15 mM isopropyl-β-d-thiogalactoside (IPTG). After growing at 20°C for 20 h, the cells were harvested and suspended in lysis buffer (50 mM Tris–HCl pH 8.0, 400 mM NaCl, 10 mM imidazole), followed by disruption of the cells by sonication on ice. The supernatant was collected after centrifugation and loaded onto a column packed with Ni-NTA beads (Qiagen). The impurity was washed with wash buffer (50 mM Tris–HCl pH 8.0, 400 mM NaCl, 10 mM imidazole), and then the His_6_-tagged *Sa*MetRS protein was eluted with elution buffer (50 mM Tris–HCl pH 8.0, 400 mM NaCl, 200 mM imidazole). The *Sa*MetRS protein was further purified by a HiLoad 16/60 Superdex 200 pg column (GE Healthcare) in running buffer (20 mM Tris–HCl pH 8.0, 200 mM NaCl, 5% glycerol, 2 mM β-mercaptoethanol). The protein purity was assessed by SDS-PAGE. The purified protein was concentrated to 30 mg/ml and stored in storage buffer (2 mM Tris–HCl pH 8.0, 50 mM NaCl, 2 mM β-mercaptoethanol) at –80°C. For the enzyme binding and inhibition assays, full-length *Sa*MetRS (*Sa*MetRS-FL) (residues 1–657) was expressed and purified in the same manner as that for the truncated protein.

As the N-terminal GST-like domain is dispensable for the aminoacylation activity of *Hc*MetRS (31), the DNA fragments encoding a truncated *Hc*MetRS without the N-terminal part (*Hc*MetRS-dN) (residues 221–900) and another truncated *Hc*MetRS without both N- and C-terminal parts (*Hc*MetRS-dN/C) (residues 221–834) (32) were amplified from the human cDNA library and inserted into the pET20b plasmid. The truncated *Hc*MetRSs were fused with an N-terminal His_6_-SUMO tag and expressed the same way as for *Sa*MetRS. Proteins were purified using a Ni-NTA column. The nonspecific binding contaminants were washed away with wash buffer (50 mM Tris–HCl pH 8.0, 400 mM NaCl, 10 mM imidazole). His_6_-tagged Ulp1 (the SUMO protease) was added to the column to digest the His_6_-SUMO tag from *Hc*MetRS-dN and *Hc*MetRS-dN/C proteins at 4°C overnight, and then non-tagged proteins were eluted with wash buffer and further purified using size-exclusion chromatography.

### Protein crystallization, diffraction data collection and structure determination

Crystallization assays were performed at room temperature using the sitting-drop vapor-diffusion method. To grow the cocrystals of *Sa*MetRS (which refers to *Sa*MetRS-dC in crystallization unless otherwise indicated) in complex with different ligands, the protein was preincubated with 5 mM of each ligand at 4°C for 30 min before the crystallization drops were set up. Each crystallization drop consisted of 1 μl of purified *Sa*MetRS (10–20 mg/ml) and 1 μl of reservoir solution and was equilibrated against 100 μl of reservoir solution at room temperature for 2–5 days to allow the crystals to grow. The crystals were grown in reservoir solutions as follows: 0.1 M Tris pH 7.5, 22% (w/v) PEG 3,350 for the apo protein; 0.1 M sodium cacodylate pH 6.3–6.7, 20–22% w/v PEG 3350 for the *Sa*MetRS·L-Met, *Sa*MetRS·ATP, *Sa*MetRS·P80 and *Sa*MetRS·fragment·ATP complexes; 0.05 M magnesium acetate, 0.1 M sodium cacodylate pH 6.6, 22% (w/v) PEG 3350 for the *Sa*MetRS·P21·ATP complex. These crystals were immersed in a cryoprotectant solution (reservoir solution supplemented with 20% ethylene glycol) for a few seconds and then flash-frozen in liquid nitrogen.

The diffraction data of apo *Sa*MetRS, *Sa*MetRS in complex with L-Met, *Sa*MetRS in complex with ATP, *Sa*MetRS in complex with fragment M3-88 and ATP and *Sa*MetRS in complex with compound P21 and ATP were collected on an in-house Rigaku Oxford Diffraction Xcalibur Nova single-crystal diffractometer with a wavelength of 1.5418 Å at 100 K and were integrated and scaled using CrysAlis^Pro^ software (Agilent Technologies UK Ltd). The diffraction data of *Sa*MetRS in complex with fragment M2-80 and ATP, *Sa*MetRS in complex with fragment M3-146 and ATP, *Sa*MetRS in complex with fragment B54 and ATP and *Sa*MetRS in complex with compound P80 were collected at the BL02U1 beamline at Shanghai Synchrotron Radiation Facility with a wavelength of 0.9791 Å at 100 K and processed with XDS (33). The structure was solved by molecular replacement using a *Sa*MetRS structure (PDB ID: 4QRE) as the search model in the program Molrep (34). Iterative refinements of the structure model were carried out using Coot (35) and Refmac5 (36). The stereochemical quality of the structure models was assessed by using MolProbity (37). The statistics of the data collection and structural refinement are listed in Supplementary Table S1. The coordinate and structural factors of the structures described in the paper have been deposited in the Protein Data Bank (PDB) under the accession codes 7WPJ, 7WPK, 7WPL, 7WPM, 7WPN, 7WPT, 7WPX, 7WQ0 and 7WPI.

### Fluorescence-based thermal shift assay

Ligand binding usually stabilizes the protein during the thermal denaturation process, and a tighter binder causes a larger positive shift in the protein melting temperature (*T*_m_) (38). The binding of ligands to *Sa*MetRS was evaluated by the fluorescence-based thermal shift assay as described (39). Briefly, mixtures with a final volume of 20 μl that contained 4 μg of *Sa*MetRS-FL, 4 × SYPRO orange fluorescence dye (Sigma-Aldrich) and different ligands (10 μM REP8839, 5 mM L-Met, 5 mM ATP, or 1 mM fragments) in TSA buffer (100 mM Tris pH 8.0, 150 mM NaCl) were prepared in 96-well plates. The mixtures were incubated at 25°C for 10 min and then heated from 25°C to 95°C at a rate of 1°C/min. The fluorescence intensity was recorded every 20 s by using a StepOnePlus Real-Time PCR instrument (Life Technologies). The melting curves, using the fluorescence signal as the ordinate and the temperature as the abscissa, were fitted by using StepOne™ software v2.3 to obtain the melting temperature (*T*_m_) of the protein. Triplicate assays were performed, and the average *T*_m_ values were used. The thermal stabilization of *Sa*MetRS that was caused by a ligand (a fragment, substrate or inhibitor) was termed Δ*T*_m_, and it was calculated by subtracting the *T*_m_ of *Sa*MetRS without the ligand from the *T*_m_ of *Sa*MetRS supplemented with the ligand as follows:

$$\begin{eqnarray*} \Delta {T_m} &=& {T_m}\left( {{\rm{Lig}}} \right) - {T_m}\left( {{\rm{apo}}} \right)\quad {\rm{or}} \nonumber \\ \Delta {T_m} &=& {T_m}\left( {{\rm{Lig + ATP}}} \right) - {T_m}\left( {{\rm{ATP}}} \right)\, \end{eqnarray*}$$

### Pretransfer editing assay

A pretransfer editing assay was employed to evaluate the enzymatic activity of MetRS and the inhibitory activity of fragments to this enzyme. MetRS uses ATP to misactivate the noncognate l-norleucine (L-Norleu) to produce norleucyl adenylate and pyrophosphate (PPi). Norleucyl adenylate was hydrolyzed in the active site through pretransfer editing, and PPi was hydrolyzed by pyrophosphatase (PPiase). The pretransfer editing activity of MetRS was evaluated by measuring phosphate ion (Pi) production using a malachite green assay (40,41). The assays were performed in 80 μl reaction mixtures consisting of 160 nM *Sa*MetRS-FL or *Sa*MetRS-dC or *Hc*MetRS-dN or *Hc*MetRS-dN/C, 100 μM ATP, 20 mM L-Norleu and 50 μg/ml PPiase in reaction buffer (30 mM HEPES pH 7.5, 150 mM NaCl, 30 mM KCl, 40 mM MgCl_2_, 1 mM DTT) at room temperature (42). The pyrophosphates released from the pretransfer editing reaction were decomposed into phosphates by PPiase. Then, 20 μl of malachite green reagent (2.45 M sulfuric acid, 0.1% w/v malachite green, 1.5% w/v ammonium molybdate tetrahydrate, and 0.2% v/v Tween 20) was added to each reaction, and the absorbance at 620 nm was recorded using a Synergy H1 microplate reader.

To measure the inhibitory activity of compounds against *Sa*MetRS, the reactions of an 80 μl mixture consisting of 160 nM *Sa*MetRS-FL (or *Hc*MetRS-dN), 100 μM ATP, 20 mM l-norleucine, 50 μg/ml PPiase and 1 mM fragment (or inhibitor at different concentrations) in reaction buffer were incubated at room temperature for 30 min. Then, 20 μl of malachite green reagent was added to each reaction, and absorbance at 620 nm was recorded. The absorbance at 620 nm of the reaction without adding a compound (fragment or inhibitor) was A_1_, the absorbance of the reaction without adding MetRS was *A*_0_, and the absorbance of the reaction with adding a compound (fragment or inhibitor) was *A*_C_. The inhibitory rates of compounds on enzyme activity = (*A*_1_ – *A*_C_)/(*A*_1_ – *A*_0_) ×100%. For compounds P80, P21 and REP8839, the inhibitory rates were measured at various concentrations, and the data were fitted in GraphPad Prism 8 using the ‘log[inhibitor] versus response’ equation to calculate the IC_50_ values.

### tRNA-dependent ATP consumption assay

The inhibitory activity of the compounds against the tRNA aminoacylation reaction of MetRS was evaluated by employing an ATP consumption assay (43,44). In brief, 10 μl reactions consisting of 50 nM *Sa*MetRS-FL or 100 nM *Hc*MetRS-dN, 4 μM ATP, 500 μM L-Met, 0.5 mg/ml *E. coli* total tRNA in reaction buffer (30 mM HEPES pH 7.5, 150 mM NaCl, 30 mM KCl, 40 mM MgCl_2_, 0.1% BSA and 1 mM DTT) and compounds (P80, P21 and REP8839) at various concentrations were prepared and incubated at room temperature for 30 min. The reactions were then stopped by adding 10 μl of Kinase-Glo™ Reagent (Promega) and incubated at room temperature for 15 min. The luminescence, which reflects the concentration of the remaining ATP, was read on a Synergy H1 microplate reader (BioTek). The luminescence of the reaction without adding a compound was Lum_1_, the absorbance of the reaction without adding MetRS was Lum_0_, and the luminescence of the reaction with the added compound was Lum_C_. The inhibitory rates of a compound on enzyme activity = (Lum_C_ – Lum_1_)/(Lum_0_ – Lum_1_) × 100%. The data were fitted in GraphPad Prism 8 with the ‘log[compound] versus response’ equation to calculate the IC_50_ values.

### Isothermal titration calorimetry assay

The affinities of ligands to *Sa*MetRS were measured by using a MicroCal PEAQ-ITC microcalorimeter. Specifically, to test the affinity of fragment M3-88 to *Sa*MetRS and the impact of AMP or ATP on the affinity, 2 mM M3-88 was titrated to 100 μM *Sa*MetRS (200 μl, alone or preincubated with 5 mM AMP or ATP) in ITC buffer (100 mM HEPES pH 7.5, 300 mM NaCl, 5% glycerol, 1% DMSO). To test the affinity of ATP to *Sa*MetRS and the impact of M3-88 on the affinity, 500 μM ATP was titrated to 50 μM *Sa*MetRS (alone or preincubated with 5 mM M3-88). To test the affinity of fragment T2-15 to *Sa*MetRS and the impact of ATP on the affinity, 2 mM T2-15 was titrated to 50 μM *Sa*MetRS (alone or preincubated with 5 mM ATP). The titration was performed at 25°C, with 0.2 μl for the first injection and 2 μl for the next 19 injections. The interval between two injections was 150 s. The disassociation constants (*K*_d_) were determined by fitting the calorimetric data to a one-site binding model by using MicroCal PEAQ-ITC analysis software.

## RESULTS

### Structures of *Sa*MetRS in the apo and substrate-binding forms

**Figure 1.**
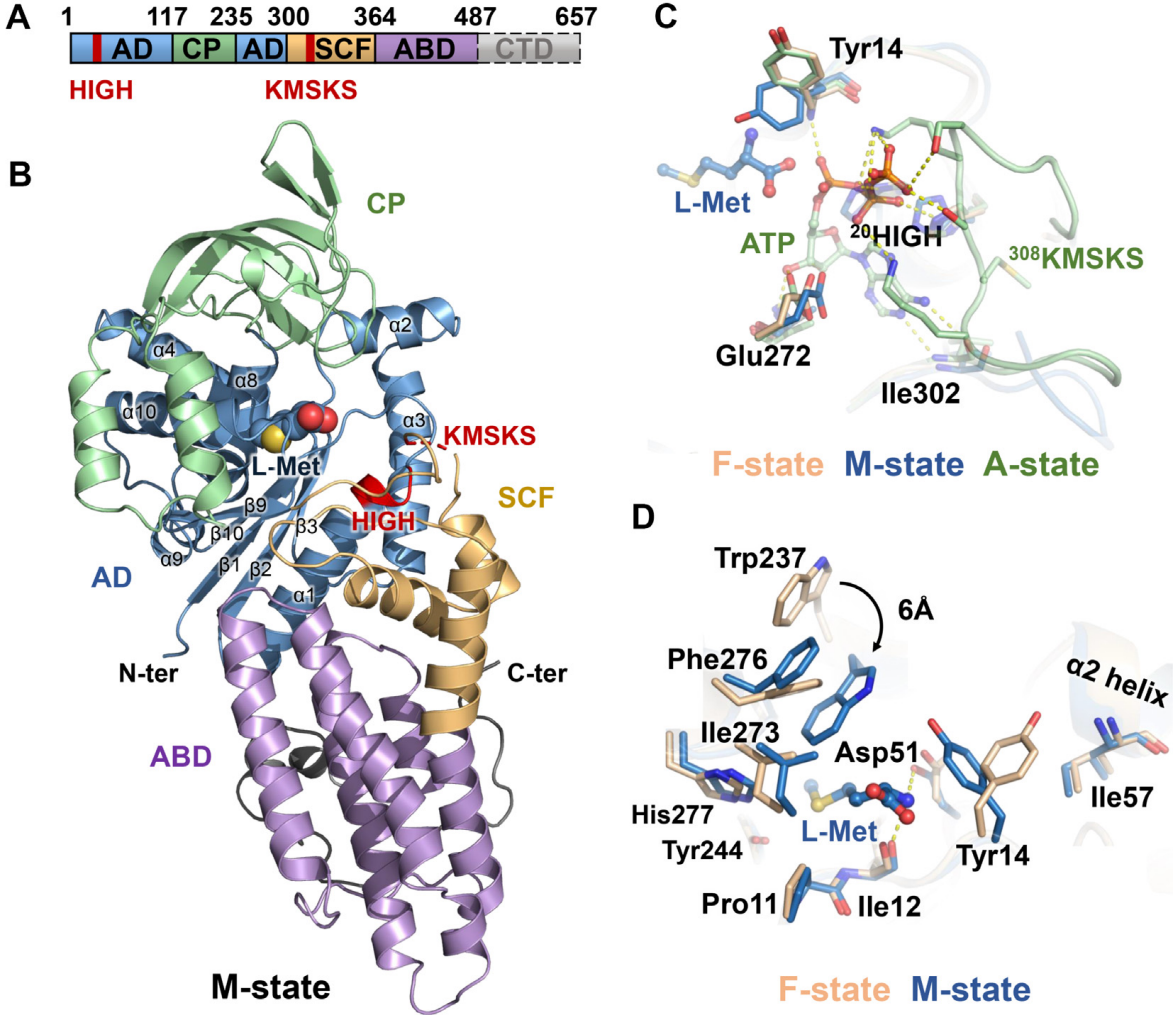
The overall structure and substrate binding modes of *Sa*MetRS. (**A**) The domain organization of *Sa*MetRS. The C-terminal domain was truncated for the crystallization assays. (**B**) The overall structure of *Sa*MetRS in its L-Met-binding form. L-Met is presented as a sphere in the active site cavity. The aminoacylation domain (AD), connecting peptide (CP), stem contact fold (SCF), anticodon binding domain (ABD) and the HIGH and KMSKS motifs are colored the same as in (A). (**C**) The interactions between ATP and ATP site residues of *Sa*MetRS. ATP stabilized the conformation of the KMSKS loop of *Sa*MetRS. (**D**) L-Met binding-induced conformational changes of resides in the L-Met site of *Sa*MetRS compared to the F-state structure. In (C) and (D), ligands (ATP and L-Met) are presented as ball-and-stick models, and structures of *Sa*MetRS in the F-state, M-state and A-state are colored in wheat, blue and green, respectively. F-state, M-state and A-state refer to the free apo form, L-Met-binding form and ATP-binding form of *Sa*MetRS, respectively.

For the crystallographic studies, a truncated form of *Sa*MetRS lacking a major part of its C-terminal domain (CTD) (*Sa*MetRS-dC, residues 1–520) was produced (Figure 1A) (45). The crystal structures of *Sa*MetRS (which refers to *Sa*MetRS-dC in crystal structure analysis unless otherwise indicated) were solved in its free apo form (F-state), L-Met-binding form (M-state) and ATP-binding form (A-state) at 2.65, 2.80 and 2.50 Å, respectively (Supplementary Table S1 and Figure S1A, B). The overall structure of *Sa*MetRS resembles that of MetRS from other organisms (10), and it consists of a Rossmann fold aminoacylation domain (AD, residues 1–116 and 235–299), a connecting peptide (CP) domain (residues 117–234) inserted into the Rossmann fold, a stem contact fold (SCF) (residues 300–363), and a helical anticodon-binding domain (ABD) (residues 364–486) (Figure 1B). The Rossmann fold, which consists of seven α-helixes (α1–α4 and α8–α10) and a five-stranded parallel β-sheet (β1–β3 and β9–β10), forms a classic catalytic core of class I AARSs and holds the first signature HIGH motif of class I AARSs. The CP domain of *Sa*MetRS contains one single knuckle that lies on the top of the catalytic core, characterizing *Sa*MetRS as MetRS1. This knuckle structure does not contain a zinc-finger motif, which is observed in both MetRS1 and MetRS2 proteins and important for catalytic activity (46,47); therefore, *Sa*MetRS can be further grouped into the family D MetRSs (11). The SCF carries a KMSKS sequence, the second signature motif of class I AARSs, which plays a conserved role in ATP binding and links ABD to AD, possessing both catalytic and structural functions.

The adenine and ribose moieties of ATP are buried in a pocket that is mainly composed of Gly22, Ser26, Ala270, Glu272, His299, Gly300, Trp301, Ile302, Lys308 and Met309. At the A-state, the KMSKS loop is in a closed conformation, and together with the HIGH motif, the loop forms intensive interactions with the phosphate groups of ATP (Figure 1C). In contrast, the electron density of the KMSKS loop is too poor to trace in both the F- and M-states, indicating its dynamic nature in the absence of ATP.

In the *Sa*MetRS·L-Met complex, the hydrophobic cavity formed by residues Pro11, Trp237, Ala240, Leu241, Tyr244, Ile273, Phe276 and His277 accommodates the side chain of L-Met. In addition, the amino moiety of L-Met forms hydrogen bonds with the backbone oxygen of Ile12 and the carboxylic group of Asp51 (Figure 1D). Interestingly, the L-Met binding pocket of *Sa*MetRS undergoes marked conformational changes upon L-Met binding. Residues Trp237 and Tyr14 rotate inwards to enclose the pocket. Notably, while L-Met binding was found to induce a similar movement for the corresponding tryptophan residue (Trp518) in *Hc*MetRS, the conformation of Tyr274 of *Hc*MetRS (corresponding to Tyr14 of *Sa*MetRS) remains the same as in the F-state (32). Tyr274 in *Hc*MetRS is hydrogen bonded to Thr318, a residue conserved only in eukaryotic cytosolic MetRS proteins (Supplementary Figure S2), which causes Tyr274 to adopt an open conformation in both the F- and M-states (PDB ID: 5GL7 and 5GOY) (Supplementary Figure S3D) (32). Uncovering the structural differences between *Sa*MetRS and its human counterpart would facilitate the rational design of selective *Sa*MetRS inhibitors.

### CP domain movements upon L-Met binding in MetRS1

**Figure 2.**
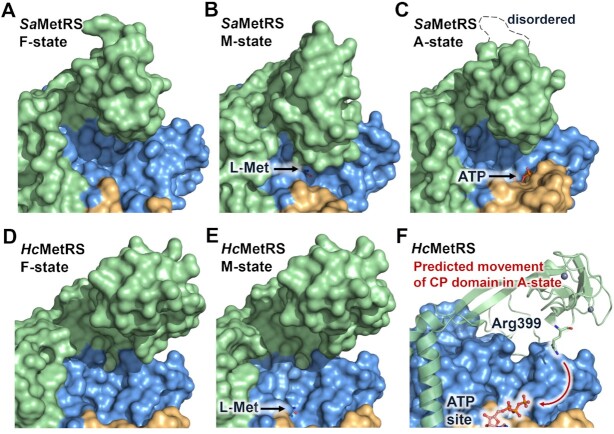
Conformational movements of the CP domain in MetRS upon substrate binding. (**A–C**) Conformations of the CP domain in the crystal structures of free (F-state), L-Met binding (M-state), and ATP binding (A-state) forms of *Sa*MetRS. (**D, E**) Conformations of the CP domain in the crystal structures of *Hc*MetRS in the F-state and M-state. (**F**) Predicted CP domain movement upon ATP binding in *Hc*MetRS. The different domains of MetRS are colored the same as in Figure 1A.

The CP domain was reported to play crucial roles in L-Met activation and the correct positioning of the CCA end of tRNA (10,47). An interesting facet of *Sa*MetRS is the large conformational movement of the CP domain. Compared to other states, the knuckle structure in the CP domain of *Sa*MetRS bends inward at the M-state, resulting in a more compact and partially covered active site cavity (Figure 2A–C). To date, there are two other M-state structures available for MetRS1 proteins, including *Tb*MetRS and *Brucella melitensis* MetRS (*Bm*MetRS). Although their apo form structures have not been determined, compared to their inhibitor-bound structures, the L-Met-induced CP domain movements of both *Tb*MetRS and *Bm*MetRS are consistent with *Sa*MetRS (Supplementary Figure S3A-C) (29,48). In contrast, L-Met binding causes few conformational changes, if any, of the CP domain in the structures of MetRS2 proteins, such as *Hc*MetRS (Figure 2D-E and Supplementary Figure S3D), *Escherichia coli* MetRS (*Ec*MetRS) (Supplementary Figure S3E) and *Xanthomonas citri* MetRS (*Xc*MetRS) (Supplementary Figure S3F) (30,32,49).

**Figure 3.**
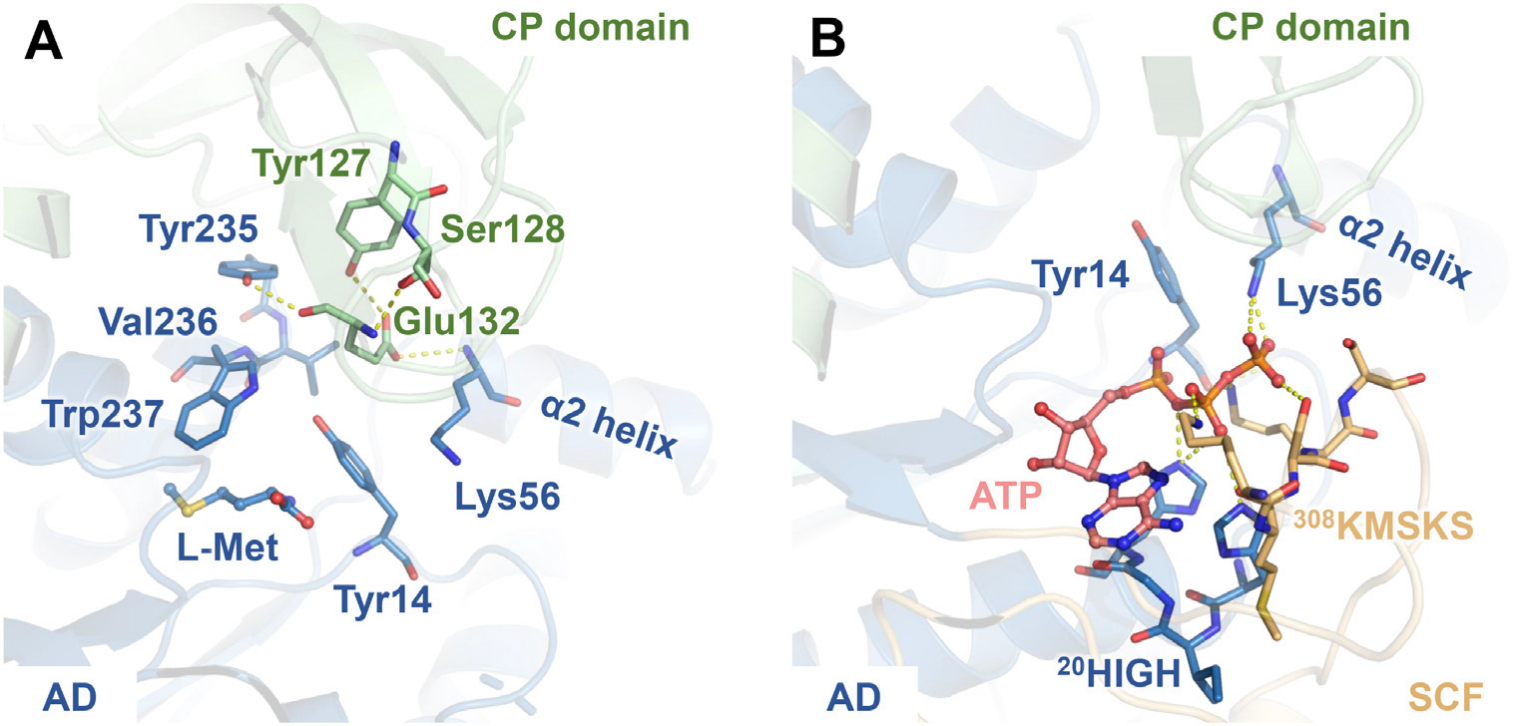
Key residues involved in stabilizing different CP domain conformations upon ligand binding. (**A**) The hydrogen-bonding network formed by residues from the aminoacylation domain and CP domain observed in the structure of the *Sa*MetRS·L-Met complex. The substrate L-Met is colored blue. (**B**) Lys56 interacts with the pyrophosphate group of ATP in the structure of *Sa*MetRS in complex with ATP (pink) and a fragment (M3-146, omitted). The different domains of *Sa*MetRS are colored the same as in Figure 1A.

We noticed that when L-Met bound to *Sa*MetRS, the indole group of Trp237 flipped downward by approximately 6 Å to form hydrophobic contacts with the side chain of L-Met (Figure 1D). The large conformational rearrangements of Trp237 and its neighboring residues, such as Tyr235, provided space for the downward movement of the CP domain. Importantly, the residue Glu132 extended to the N-terminal of the α2 helix on the Rossmann fold, forming an ion-dipole interaction that contribute to trapping the CP knuckle in its bent conformation (Figure 3A). While Tyr235, Val236 and Trp237 are strictly conserved in MetRS from different organisms, residue Glu132 is only conserved in MetRS1 but not MetRS2 (Supplementary Figure S2). The corresponding residue in MetRS2 is Lys, Gly, Asn, Gln or Ala, which cannot form a similar ion-dipole interaction with the Rossmann fold. This may explain why the CP domain in MetRS2 does not adopt a bent conformation to form a more compact and partially covered active site cavity when L-Met binds.

In contrast, a positively charged Arg399 in the CP knuckles of *Hc*MetRS has been suggested to contribute to stabilizing ATP or the pyrophosphate released after the methionyl adenylate intermediate is formed, implying that CP knuckles bend downward to interact with ATP after ATP binding (Figure 2F) (32). However, ATP-bound structure is not available for MetRS2 thus far. Arg399 in *Hc*MetRS is completely conserved in all MetRS2 proteins but is missing in all MetRS1 proteins (Supplementary Figure S2). While the CP knuckle surface that faces the ATP site is positively charged in MetRS2, in contrast, this surface is mainly negatively charged in MetRS1 (Supplementary Figure S4). Interestingly, the role of Arg399 in *Hc*MetRS seems to be performed by a conserved lysine in MetRS1 that is located at the α2 helix of the Rossmann fold, for examples, Lys56 in *Sa*MetRS and Lys261 in *Leishmania major* MetRS (*Lm*MetRS). The interaction between this lysine residue and the phosphate groups was observed in the structure of *Sa*MetRS in complex with ATP and two chemical fragments (fragments M3-146 and B54, see below) (Figure 3B) as well as the structure of *Lm*MetRS in complex with a methionyl adenylate and a pyrophosphate (PPi) (PDB ID: 3KFL) (50). Therefore, the CP knuckle of MetRS1 has fewer interactions with ATP compared to that of MetRS2, and as a result, the CP knuckle maintains a similar open conformation in both the F- and A-states.

Thus, our structural analysis suggested that substrate-induced conformation movements of the CP domain of *Sa*MetRS are distinct from those of *Hc*MetRS due to the sequence difference of the CP domain in the two proteins.

### Fragment screening against *Sa*MetRS

**Figure 4.**
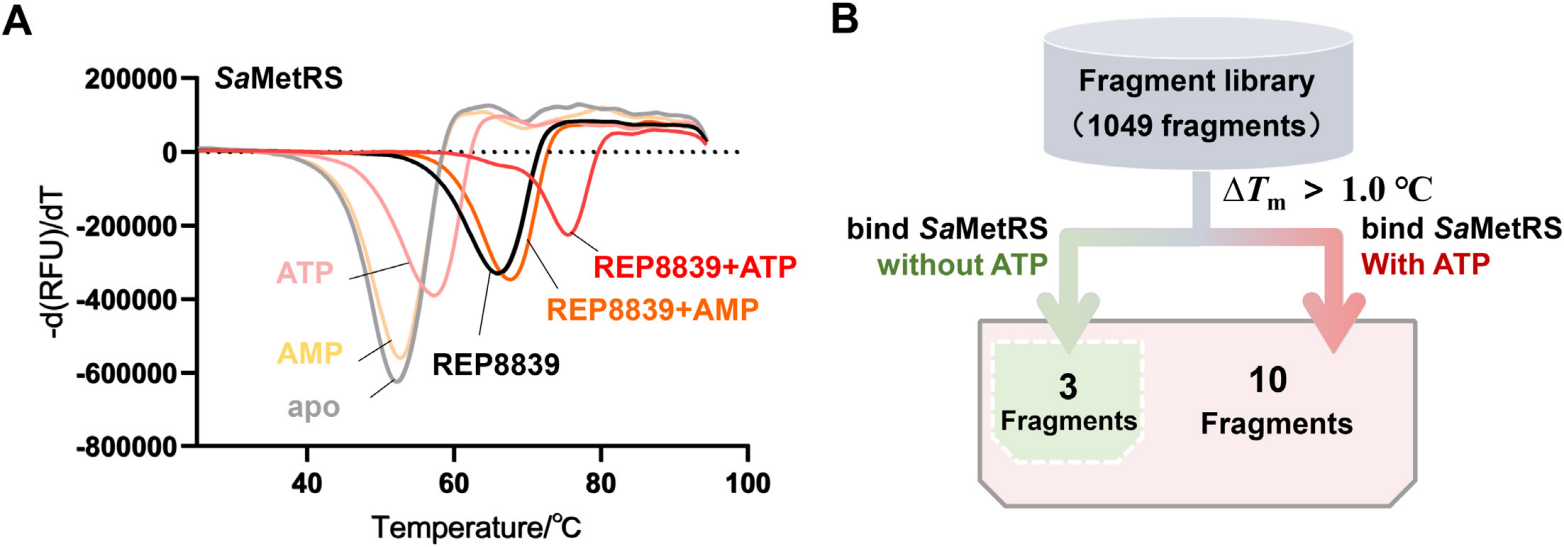
ATP-assisted fragment screening against *Sa*MetRS by using TSA. (**A**) Thermal melting curves of *Sa*MetRS in the presence of different ligands and their combinations. ATP could further shift the *T*_m_ of the *Sa*MetRS supplemented with REP8839. (**B**) Schematic illustration of the TSA-based fragment screening process. A fragment was identified as a binder of *Sa*MetRS if it increased the *T*_m_ value of *Sa*MetRS by >1.0°C. Fragments were screened in parallel against both the apo *Sa*MetRS and *Sa*MetRS supplemented with ATP. Ten fragments were identified to bind the *Sa*MetRS supplemented with ATP, including three fragments that could bind the *Sa*MetRS without ATP.

In the complex structure of *Sa*MetRS with ATP, the CP domain adopts a relatively open conformation, similar to that of the conformation of the CP domain when REP8839 binds (30). In addition, REP8839 was reported to exhibit improved inhibitory activity at higher ATP concentrations (25), a behavior opposite to most AARS inhibitors. We then examined the binding affinity of REP8839 to *Sa*MetRS (which refers to *Sa*MetRS-FL in all the biophysical and biochemical assays unless otherwise indicated) by employing fluorescence-based TSA. Specifically, ligand-free apo protein and proteins saturated with ATP or AMP represented the F-, A- and A'-state, respectively. The results showed that REP8839 caused a significantly greater *T*_m_ shift of *Sa*MetRS in the A-state (Δ*T*_m_ = 18.1°C) than in the F-state (Δ*T*_m_ = 13.6°C) and A'-state (Δ*T*_m_ = 15.4°C), revealing the synergistic effect of ATP on the binding of REP8839 to *Sa*MetRS (Figure 4A).

Fragment screening is a fast-growing strategy for designing drugs with novel scaffolds as well as for identifying new druggable sites (51,52). A total of 1049 fragments or low molecular weight natural products were screened by using TSA to search for new binders to *Sa*MetRS. Considering the potential beneficial effect of ATP on ligand binding to *Sa*MetRS, a parallel experiment was carried out using *Sa*MetRS, which was saturated with 5 mM ATP. Fragments were diluted to a final concentration of 1.0 mM. The *T*_m_ of apo or ATP-bound *Sa*MetRS without adding fragments was used as a blank control, and REP8839 was used as a positive control. We considered fragments that could increase the *T*_m_ value of *Sa*MetRS by more than 1.0°C (Δ*T*_m_ > 1.0°C) as positive hits (Figure 4B).

Finally, a total of ten hits were identified. Except for fragments T2-15 and A6, the Δ*T*_m_ values of the other eight fragments to *Sa*MetRS were significantly improved by ATP, and the binding of seven fragments could only be detected in the presence of ATP (Table 1), strongly supporting our ATP-assisted strategy.

**Table 1.** Δ*T*_m_ and inhibitory rates of the fragments identified against *Sa*MetRS

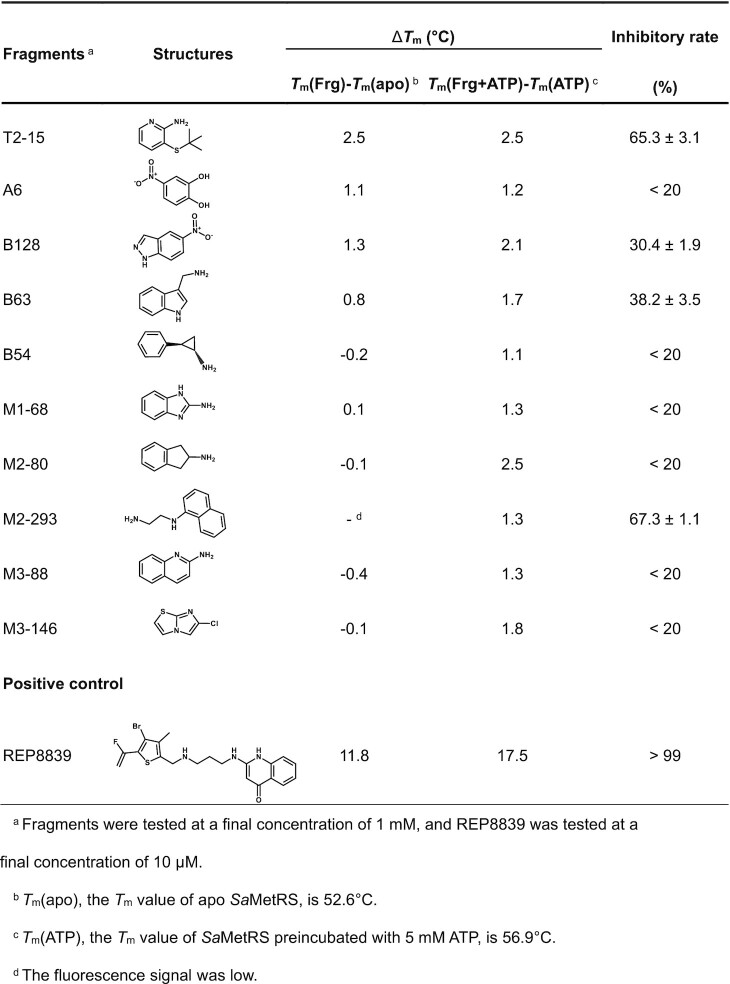

The inhibitory rates of these fragments against *Sa*MetRS were then tested by a pretransfer editing assay. In the assay, MetRS used ATP to misactivate noncognate L-norleucine (L-Norleu) to produce norleucyl adenylate and PPi. The norleucyl adenylate was then quickly hydrolyzed (or ‘edited’) by MetRS in a tRNA-independent manner (53–55). In addition, PPi is hydrolyzed by pyrophosphatase, and phosphate production reflects the activity of MetRS in L-Norleu misactivation and pretransfer editing. The tRNA-independent pretransfer editing reaction for the noncognate norleucyl adenylate, whether full-length or C-terminal domain (which is involved in tRNA binding) truncated *Sa*MetRS was used, was much greater efficient than methionine activation reaction, resulting in a higher signal for measurement (Supplementary Figure S5). Inhibitory rates of fragments at 1 mM are shown in Table 1.

### Chemical fragments bound to either the L-Met site or auxiliary pocket of *Sa*MetRS

**Figure 5.**
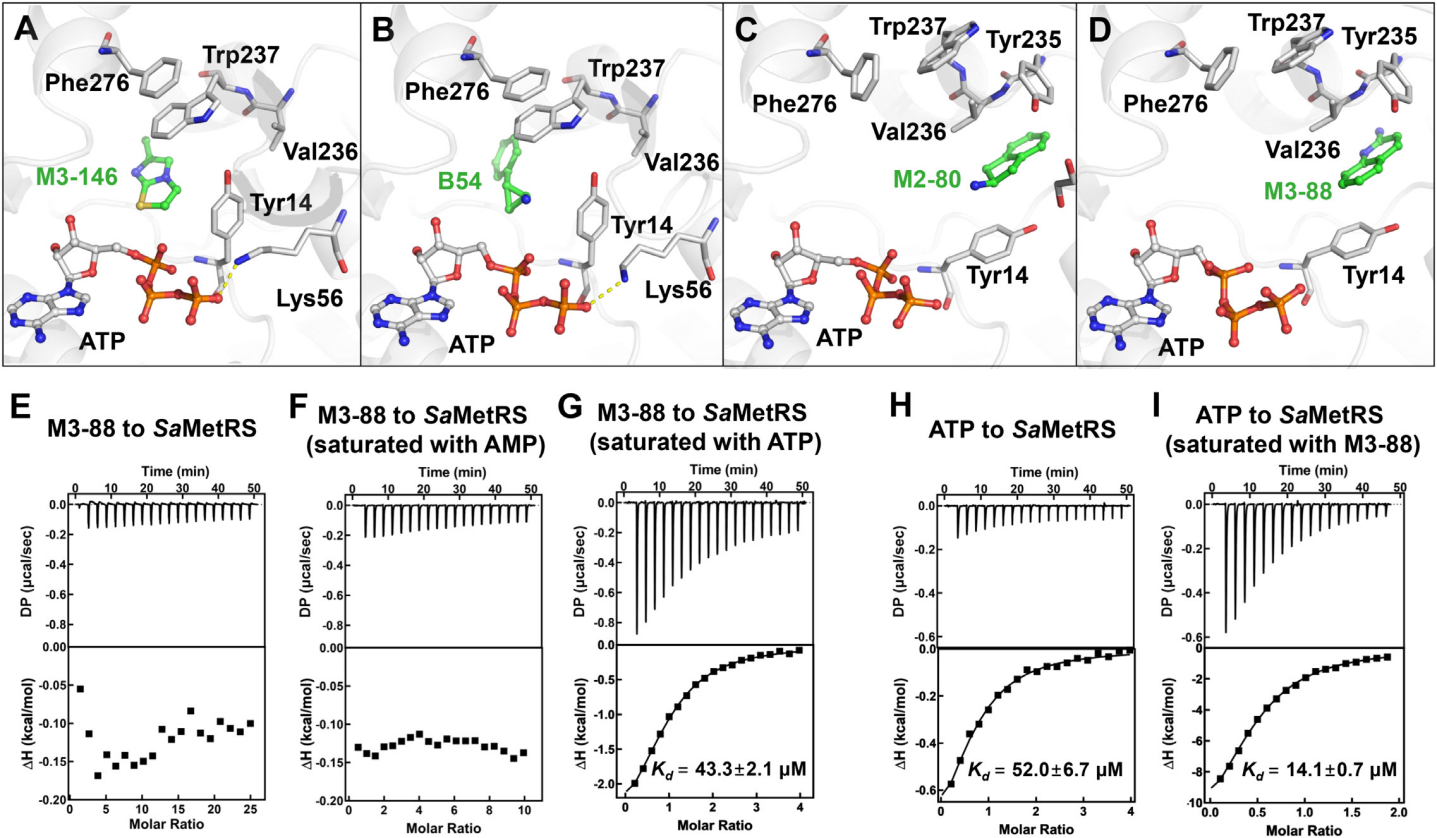
ATP-assisted fragment binding to *Sa*MetRS. (A–D) Binding modes of four fragments to *Sa*MetRS in the presence of ATP were determined by crystallography. Fragments M3-146 (**A**) and B54 (**B**) bound to the L-Met binding site of *Sa*MetRS, and fragments M2-80 (**C**) and M3-88 (**D**) bound to the auxiliary pocket of *Sa*MetRS. (E–G) ITC titrations of fragment M3-88 to *Sa*MetRS alone (**E**), saturated with AMP (**F**) and saturated with ATP (**G**). M3-88 only showed significant binding affinity in the presence of ATP. (H–I) ITC titrations of ATP to *Sa*MetRS alone (**H**) and saturated with M3-88 (**I**) showed that M3-88 also improved the binding of ATP to *Sa*MetRS.

The binding modes of the four fragments to *Sa*MetRS have been clarified by cocrystal structures (Supplementary Table S1). Two fragments (M3-146 and B54) occupied the L-Met binding site, and the other two fragments (M2-80 and M3-88) occupied the auxiliary pocket (Figure 5A–D and Supplementary Figure S1C–F). ATP was found to co-bind in the active site cavity with all four fragments.

Previous studies have shown that when diaryldiamine-based inhibitors bind to the L-Met pocket of *Tb*MetRS, the pocket remains in an open conformation similar to that in the F-state. In contrast, fragments M3-146 and B54 are surrounded by residues Tyr14, Trp237 and Phe276, which undergo conformational changes to enclose the L-Met site (Figure 5A, B). Thus, M3-146 and B54 partially mimic the binding of the substrate L-Met. Moreover, ATP has been co-crystallized in the structures, so the ternary complexes are supposed to close to an intermediate state in the amino acid activating process. Notably, in this state, ATP forms hydrogen bonds with residue Lys56 (Figures 3B and 5A, B), which has not been observed in the binary complex (*Sa*MetRS·ATP) structure.

Two fragments, M2-80 and M3-88, were found to bind to an auxiliary site in the active site cavity (Figure 5C, D). Although there are no direct conflicts between these two fragments and the substrate L-Met, both fragments block the movement of the CP domain as well as the conformational changes of the key residues required for L-Met binding (Supplementary Figure S6A). Moreover, structure superimposition of the *Sa*MeRS·fragment·ATP and *Ec*LeuRS·tRNA^Leu^ complex (PDB ID: 4AQ7) suggests that fragments M3-88 and M2-80 are located near the CCA end binding site of *Sa*MetRS and conflict with nucleotide A76 of tRNA (Supplementary Figure S6B). Thus, it is speculated that the auxiliary site fragments could inhibit the functional binding of substrates L-Met and tRNA^Met^ to *Sa*MetRS in an allosteric and orthosteric manner, respectively.

In contrast, all the fragments identified in this work do not seem to compete with ATP since they further shift the *T*_m_ of *Sa*MetRS saturated with ATP by more than 1.0°C. To further investigate this hypothesis, we measured the binding affinity of fragments with *Sa*MetRS alone or *Sa*MetRS saturated with ATP/AMP by isothermal titration calorimetry (ITC). In the presence of ATP, the auxiliary site fragment M3-88 bound to *Sa*MetRS with an affinity of 43.3 ± 2.1 μM, whereas in the absence of ATP (*Sa*MetRS apo or saturated with AMP), no binding curve could be detected (Figure 5E–G). We also measured the isotherm of titrating ATP to *Sa*MetRS premixed with M3-88, and the *K*_d_ of 14.1 ± 0.7 μM was approximately 4-fold more potent than the *K*_d_ (52.0 ± 6.7 μM) of titrating apo protein (Figure 5H-I). These results clearly showed the cooperative binding of the auxiliary site fragment M3-88 and ATP to *Sa*MetRS. As a control, fragment T2-15 bound to *Sa*MetRS premixed with or without ATP with similar affinity of 54.6 ± 2.6 and 78.7 ± 7.1 μM, respectively (Supplementary Figure S7), indicating that there was no synergistic coupling between T2-15 and ATP, and this was consistent with the TSA results in which T2-15 caused similar Δ*T*_m_ values for *Sa*MetRS with or without adding ATP. On the other hand, the binding affinity of other fragments or REP8839 with *Sa*MetRS failed to be detected with or without ATP due to a low exothermic peak and protein aggregation.

### ATP-assisted binding was highlighted by a novel *Sa*MetRS inhibitor

**Figure 6.**
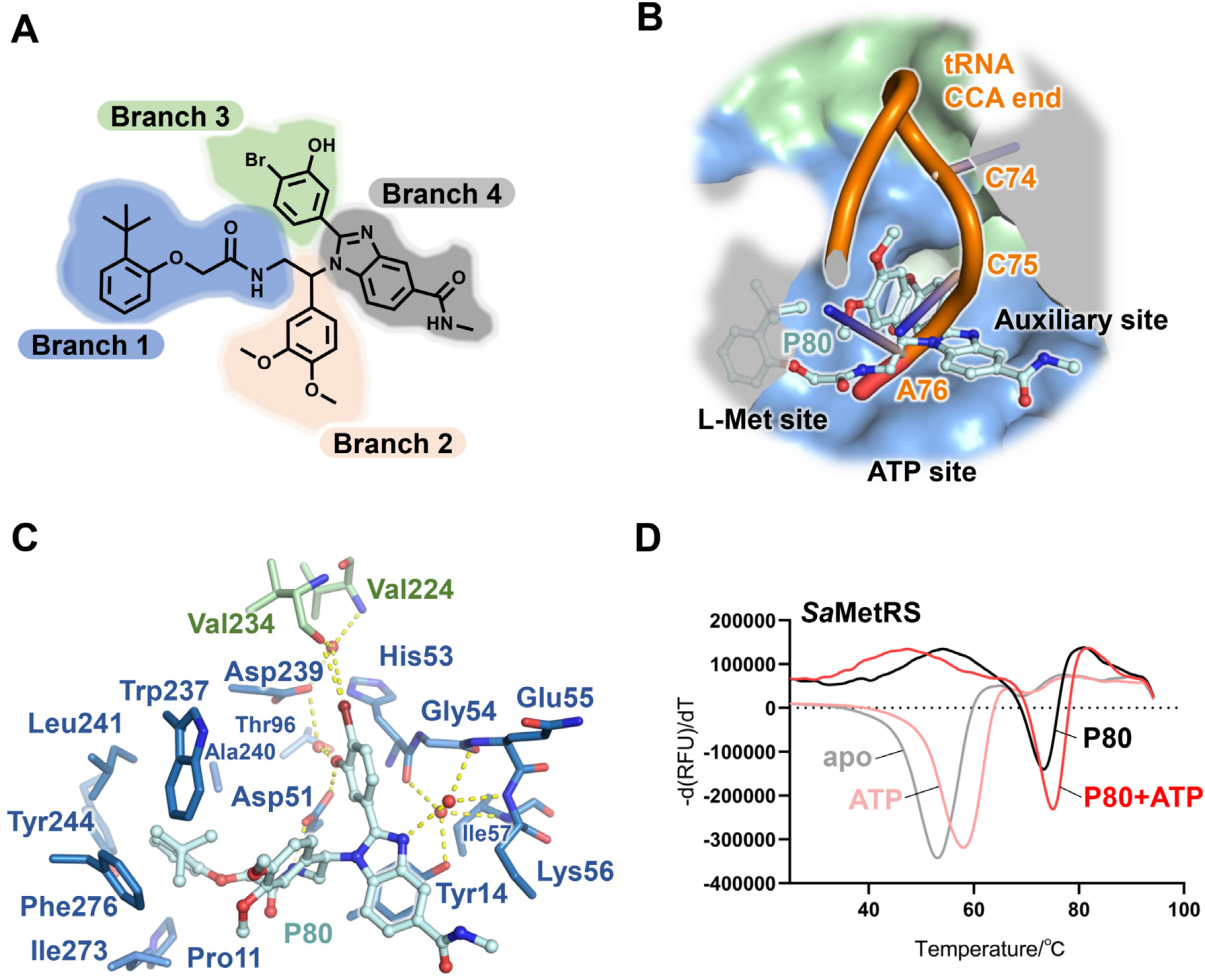
Structure of *Sa*MetRS in complex with the inhibitor P80. (**A**) Chemical structure of the phenylbenzimidazole-based compound P80. (**B**) The binding of P80 to the enlarged L-Met pocket, tRNA CCA end binding site and the auxiliary pocket. (**C**) Interactions between compound P80 and residues in *Sa*MetRS. (**D**) Thermal melting curves confirmed the strong binding of P80 to *Sa*MetRS. The largest thermal shift of *Sa*MetRS was caused by the combination of P80 and ATP, indicating the co-binding of these two chemicals.

Recently, a series of phenylbenzimidazole inhibitors against *Sa*MetRS were identified through DNA encoded library technology (DELT), and these inhibitors exhibited potent enzyme inhibition and moderate antibacterial activities (56). However, the structures of these inhibitors do not resemble any substrate or known inhibitor of MetRS (Figure 6A), making their binding mechanism unclear. A representative compound (named P80 in our study) was synthesized, and its inhibitory activity against *Sa*MetRS was measured. P80 inhibited the pretransfer editing activity of *Sa*MetRS with IC_50_ = 94.6 ± 6.7 nM (Supplementary Figure S8A). In the tRNA-dependent ATP consumption assay, which measures the overall inhibitory effects against both the first step (amino acid activation) and the second step (tRNA charging) of the aminoacylation reaction (43), P80 inhibited *Sa*MetRS with an IC_50_ value of 223.0 ± 12.3 nM (Supplementary Figure S8D). In contrast, the phenylbenzimidazole compounds did not inhibit *Hc*MetRS (Supplementary Figure S9), indicating the high selectivity of this class of inhibitors to MetRS1.

We determined the crystal structure of *Sa*MetRS with P80 at 1.92 Å resolution (Supplementary Table S1 and Figure S1G). The structure of compound P80 features four aromatic groups branching from a chiral center. As shown in its cocrystal structure with *Sa*MetRS, the four branches of P80 stretched in different directions to interact with *Sa*MetRS (Figure 6B-C). As the first branch, the tert-butylbenzene segment lies on the L-Met binding site and forms extensive hydrophobic interactions with residues Pro11, Trp237, Ala240, Leu241, Tyr244, Ile273 and Phe276. Additionally, its amino group formed a hydrogen bond with Asp51. Notably, the size of the tert-butylbenzene moiety is significantly larger than that of the substrate L-Met. Binding of this moiety to the L-Met site did not induce the Trp237 flip like what L-Met did, and maintained an open L-Met site, as observed in F- and A-state *Sa*MetRS. As a result, the CP domain did not bend, keeping the auxiliary pocket in the open conformation. As the second branch, the dimethoxybenzene group contributed little to the interactions with *Sa*MetRS. When the tRNA molecule was modeled to *Sa*MetRS according to the cocrystal structure of *E. coli* LeuRS with tRNA^Leu^ (PDB ID: 4AQ7), the dimethoxybenzene group largely overlapped with nucleotide A76 of tRNA, indicating competition with the functional binding of the biomacromolecular substrate tRNA^Met^. As the third branch, the bromobenzene moiety of P80 stretched deeply into the auxiliary pocket formed by residues Asp51, His54, Gly55, Glu55, Lys56, Ile57, Thr96 and Asp239 from AD and residues Val224 and Val234 from the CP domain. The benzimidazole group formed the fourth branch, and it extended toward the ATP phosphate group binding site.

**Figure 7.**
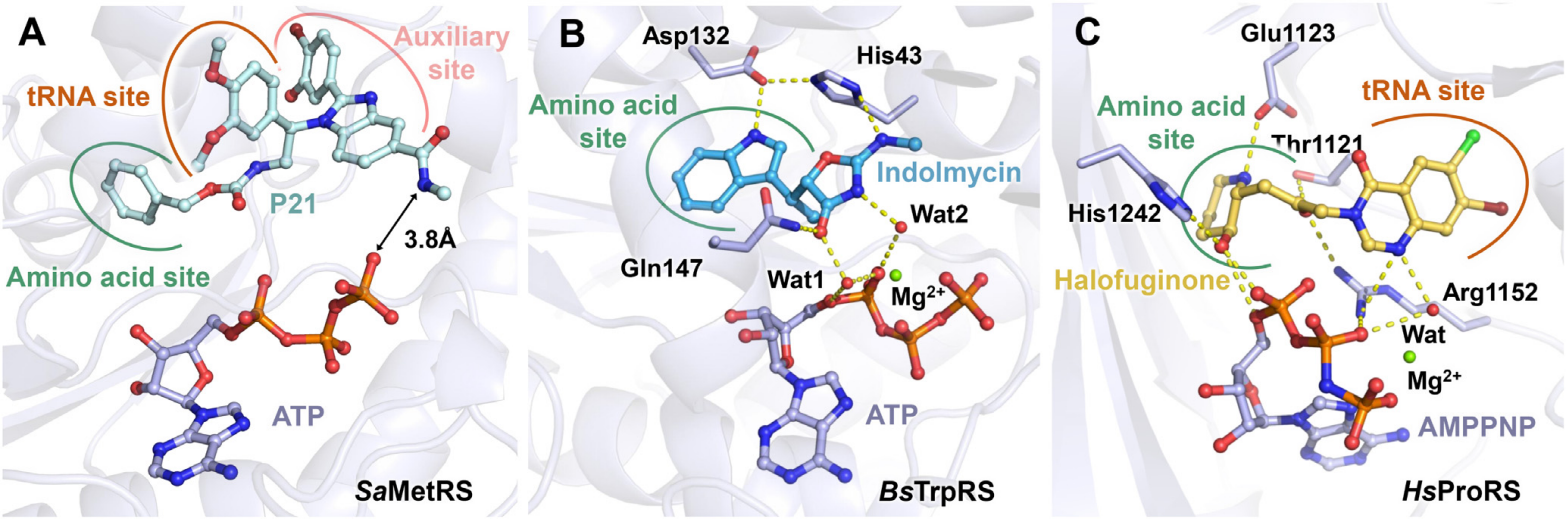
ATP-coupled binding of inhibitors to AARSs. (**A**) Structure of *Sa*MetRS in complex with the inhibitor P21 and ATP. No interaction was observed between ATP and P21 in the active site cavity of *Sa*MetRS. The closest distance between the two molecules is 3.8 Å. (**B**) Structure of *Bacillus stearothermophilus* tryptophanyl-tRNA synthetase (*Bs*TrpRS) in complex with indolmycin and ATP. Indolmycin forms water-mediated interactions with ATP in the active site cavity of *Bs*TrpRS (PDB ID: 5DK4). (**C**) Structure of *Homo sapiens* prolyl-tRNA synthetase (*Hs*ProRS) in complex with halofuginone and AMPPNP. Halofuginone forms direct and water-mediated interactions with AMPPNP in the active site cavity of *Hs*ProRS (PDB ID: 4HVC).

We evaluated the binding of compound P80 to *Sa*MetRS with or without ATP by TSA. Indeed, the largest thermal shift of *Sa*MetRS was caused by the combination of P80 and ATP, indicating that the co-binding of these two molecules occurred (Figure 6D). We then crystallized *Sa*MetRS supplemented with both ATP and an analog of P80 (named P21, with an IC_50_ of 427.9 ± 53.0 nM in the pretransfer editing assay and 3.11 ± 0.48 μM in the ATP consumption assay against *Sa*MetRS) (Supplementary Figure S8B and S8E), and the ternary complex structure was solved to 2.40 Å resolution (Supplementary Figure S1 and Table S1), which confirmed the co-binding of this series of inhibitors with ATP. Thus, our structural and biophysical results on fragments and the newly reported inhibitors all highlighted ATP-assisted ligand binding in the active site cavity of MetRS1. Similar to fragments M2-80 and M3-88, no interaction was observed between ATP and P21 bound with *Sa*MetRS (Figure 7A). The closest distance was 3.8 Å between the N atom of the methylacetamide substituent on the benzimidazole group of P21 and the O atom of the γ-phosphate group of ATP.

Interestingly, ATP-assisted ligand binding has also been observed in some other AARSs. In these cases, for example, the binding of indolmycin to tryptophanyl-tRNA synthetase (TrpRS) (Figure 7B) (57) and the binding of halofuginone to prolyl-tRNA synthetase (ProRS) (Figure 7C) (58), direct and water-mediated interactions between ATP and the inhibitors were observed. Indolmycin, a natural tryptophan analog, uses its indole ring to form a hydrogen bond with Asp132 of *Bacillus stearothermophilus* TrpRS, similar to the substrate L-tryptophan (L-Trp). In addition, compared to l-tryptophan, the additional oxazolinone group of indolmycin formed new interactions with residues His43, Gln147 and ATP, accounting for the high affinity inhibition of *Bs*TrpRS by indolmycin. Halofuginone is a dual-site (amino acid binding site and tRNA binding site) inhibitor of eukaryotic ProRS, with its piperidine ring competing with proline and the quinazolinone group mimicking the 3’-end nucleotide A76 of tRNA^Pro^. In the human ProRS·halofuginone·AMPPNP ternary complex, halofuginone formed hydrogen-bonding interactions with residues Thr1121, Glu1123, Arg1152 and His1242 and several hydrophobic contacts with other active site residues. Moreover, the phosphate groups of AMPPNP, a nonhydrolyzable analog of ATP, formed direct and water-mediated hydrogen bonds with the hydroxyl group of the hydroxypiperidine ring and the nitrogen atom of the quinazolinone ring of halofuginone. The direct and water-mediated interactions between ATP and inhibitors helped to explain how ATP facilitates the binding of these inhibitors to TrpRS and ProRS. However, in contrast, ATP-assisted ligand binding to MetRS is likely independent of the contacts between the ligand and ATP but in a more circuitous way.

## DISCUSSION

AMR is among the major worldwide public health threats in the twenty-first century (59,60). As one of the most frequent causes of skin and soft tissue infections in the world, drug-resistant *S. aureus*, like methicillin-resistant *S. aureus* (MRSA), emerges rapidly due to the imprudent usage of antibiotics (1).

MetRS is recognized as a promising target for the discovery of new antimicrobials, and MetRS inhibitors have been suggested to possess potent efficacy against a variety of gram-positive pathogens, including *S. aureus*. Nevertheless, the structure of *Sa*MetRS has not been discussed, specifically regarding its conformation features at different substrate binding states. In this work, we reported the crystal structures of *Sa*MetRS in its apo, L-Met-binding, and ATP-binding forms. We showed that L-Met binding induced a bent conformation of the CP domain of *Sa*MetRS, resulting in a more compact and partially covered active site cavity. Structural analysis suggested that Glu132 on the top of a CP knuckle loop may play a central role in stabilizing the bent conformation of the CP domain by forming an ion-dipole interaction with the α2 helix. While Glu132 is only conserved in MetRS1 but not MetRS2 proteins, it provides a possible reason why L-Met-induced CP domain bending was not observed in MetRS2 proteins such as *Hc*MetRS. The CP domain is involved in forming the auxiliary pocket; therefore, the differences in structural organizations, charge distribution and dynamic movements of CP domains between MetRS1 and MetRS2 are of great potential for developing selective MetRS1 inhibitors.

While traditional AARS inhibitors are typically amino acid and/or ATP competitors, AARS inhibitors with other unusual mechanisms are desirable (61). In particular, inhibitors with substrate- or intermediate-aided binding mechanisms usually showed superior activity or selectivity compared to that of traditional competitive inhibitors. Therein, the ProRS inhibitor halofuginone and the TrpRS inhibitor indolmycin represent the predominant examples of ATP-assisted inhibitors of AARSs (57,58). Since ATP molecules usually reach millimolar concentrations in bacterial cells, antibacterial agents possessing ATP-assisted inhibitory mechanisms may have better efficacy in vivo.

In our study, premixing *Sa*MetRS with ATP increases the binding affinity of REP8839, indicating that it has the potential to develop *Sa*MetRS inhibitors with the assistance of ATP. Our efforts on fragment screening set an example of searching for inhibitors that share an ATP-assisted binding mode. A significantly higher hit rate of the screening was achieved in the presence of ATP than in the absence of ATP. ITC data confirmed that fragment M3-88 achieved higher affinity in the presence of ATP and *vice versa*, indicating synergies between the two ligands on *Sa*MetRS binding. Crystallographic studies proved that these ATP-assistant fragments could bind to both the L-Met site (fragments M3-146 and B54) and the auxiliary pocket (fragments M2-80 and M3-88). Evidently, premixing *Sa*MetRS and AMP had fewer impacts than that of ATP on the binding affinity of fragments (and REP8839) to *Sa*MetRS, prompting us that the pyrophosphate group of ATP may account for the synergistic effect on inhibitors.

However, in contrast to the mechanism in ATP-assisted binding of indolmycin to TrpRS and halofuginone to ProRS, we did not observe either a direct or water-mediated interaction between ATP and fragments or between ATP and the phenylbenzimidazole inhibitor P21. The underlying reasons for the assistance of ATP in fragment and inhibitor binding with *Sa*MetRS are still not clear. The dynamics of the CP domain resulted in exchange between the open and closed conformations of the active site cavity of MetRS. While the open conformation benefits the ligand entering the active site cavity, the closed active site is required for L-Met activation (29,32,50). Previous studies suggested that the binding of ATP induces a closed conformation in MetRS2 (32). In contrast, our study showed that it is not ATP but L-Met that induces the closed conformation in *Sa*MetRS (belonging to MetRS1). Moreover, due to the negatively charged surface of the CP knuckle (Supplementary Figure S4), the phosphate groups of ATP may cause repulsion with the CP domain. Thus, according to the conformational selection mechanism (29,62,63), the binding of ATP (when no L-Met is bound in the active site) may enrich the preexisting relatively open conformations of *Sa*MetRS, which are preferable for fragments or inhibitors to enter the active site cavity. If this hypothesis is correct, ATP-assisted ligand binding will not be applicable to MetRS2, to which ATP binding induces the closed conformation. Future studies, such as long-term molecular dynamics (MD) simulations, may be helpful to elucidate the mechanism of ATP-assisted ligand binding for MetRS1. On the other hand, introducing chemical groups that form direct interactions with ATP could be a promising strategy to improve the binding affinity and species selectivity for these *Sa*MetRS inhibitors.

In conclusion, we determined nine crystal structures of *Sa*MetRS as a representative MetRS1 in apo form or in complex with different substrates, chemical fragments and inhibitors. The structural and experimental data both suggested that ATP could play an assistant role in the binding of inhibitors to the amino acid site, auxiliary site and possibly also tRNA site in the active cavity of *Sa*MetRS. In addition, the fragments we identified in the fragment screening assay also provide useful building blocks for developing drug-like inhibitors against *Sa*MetRS and even other MetRS1 proteins.

## DATA AVAILABILITY

Atomic coordinates and structure factors for the reported crystal structures have been deposited with the Protein Data Bank under accession number 7WPJ, 7WPK, 7WPL, 7WPX, 7WQ0, 7WPM, 7WPT, 7WPI, 7WPN.

## Supplementary Material

gkac285_Supplemental_File
