# Supplementary material for: Fragment screening and structural analyses highlight the ATP-assisted ligand binding for inhibitor discovery against type 1 methionyl-tRNA synthetase
Source: Nucleic Acids Res. 2022 Apr 26;50(8):4755–68. doi: 10.1093/nar/gkac285 (PMC9071491; doi:10.1093/nar/gkac285)
Supplement: gkac285_Supplemental_File [file gkac285_supplemental_file.pdf]

Supplementary Information

Fragment screening and structural analyses highlight the ATP-assisted ligand binding for inhibitor discovery against type 1 methionyl-tRNA synthetase

Jia Yi[1,2,#], Zhengjun Cai[1,2,#], Haipeng Qiu[1,2], Feihu Lu[1,2], Zhiteng Luo[1,2], Bingyi Chen[1,2], Qiong Gu[2], Jun Xu[2], Huihao Zhou[1,2,*]

[1]Guangdong Provincial Key Laboratory of Chiral Molecule and Drug Discovery, School of Pharmaceutical Sciences, Sun Yat-sen University, Guangzhou 510006, China

[2]Research Center for Drug Discovery, School of Pharmaceutical Sciences, Sun Yat-sen University, Guangzhou 510006, China

[#]These authors contributed equally to this work

* To whom correspondence should be addressed. Tel: +86 20 39943350; Email: zhuihao@mail.sysu.edu.cn

Supplementary Figures

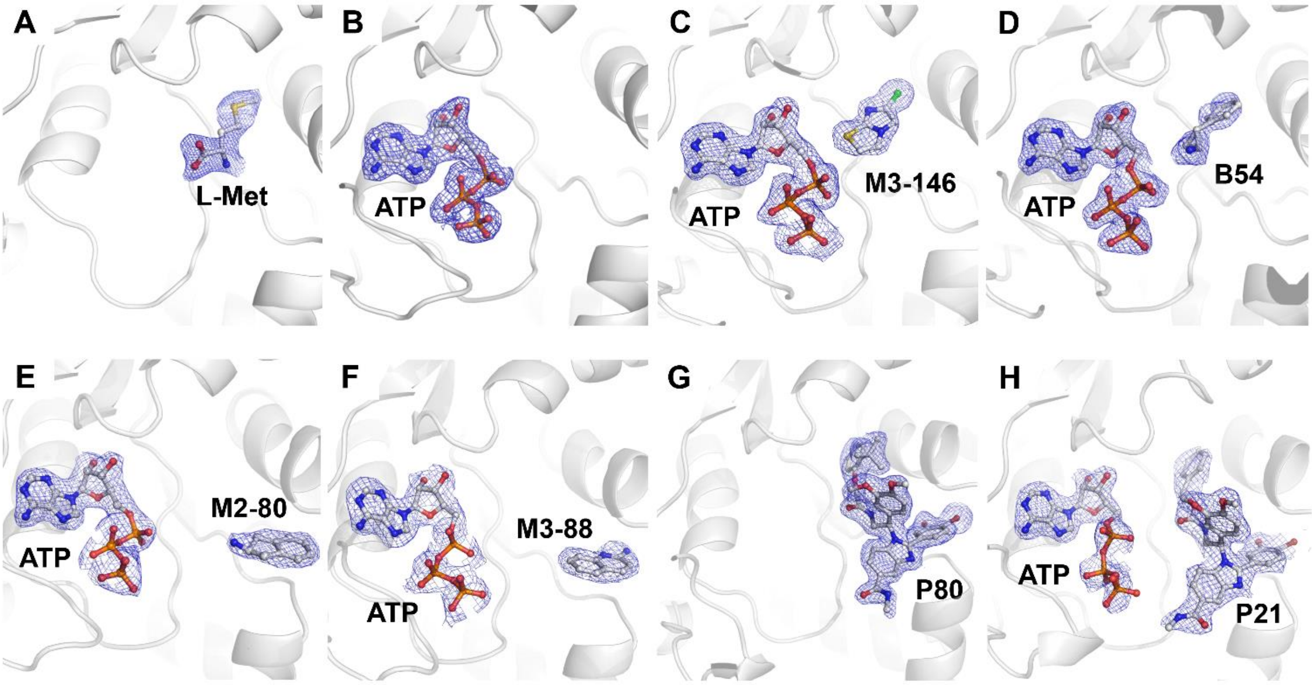

Supplementary Figure S1. 2Fo–Fc omit electron density maps of ligands bound to *Sa*MetRS. The omit maps were drawn as blue meshes contoured at 1.0 σ.

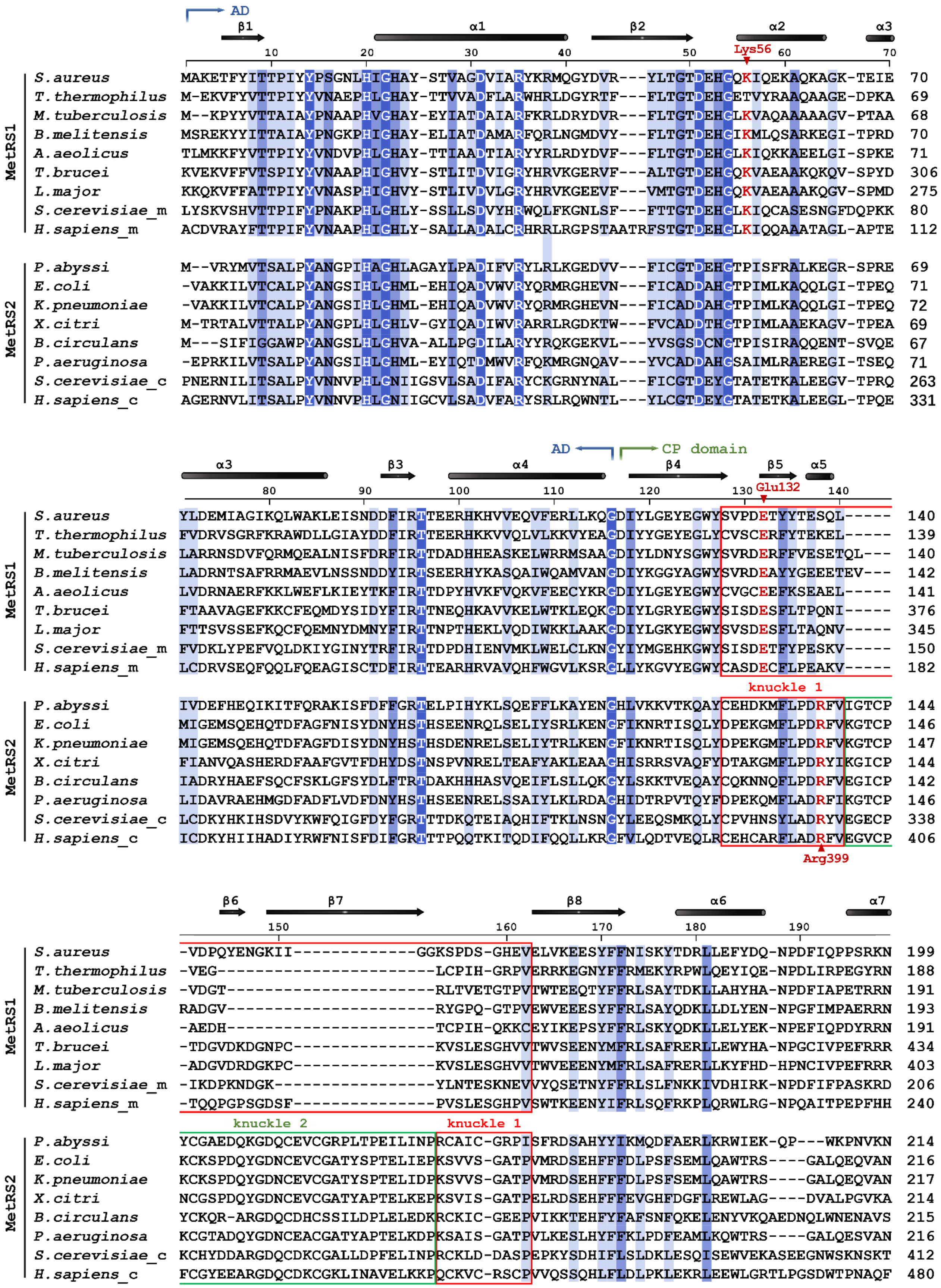
AD
β1 α1 β2 α2 α3
10 20 30 40 50 60 70
Lys56
MetRS1
S.aureus MAKETFYITTPIYYPSGNLHIGHAY-STVAGDVIARYKRMQGYDVR---YLTGTDEHGQKIQEKAQKAGK-TEIE 70
T.thermophilus M-EKVFYVTTPIYYVNAEPHLGHAY-TTVVADFLARWHRLDGYRTF---FLTGTDEHGETVYRAAQAAGE-DPKA 69
M.tuberculosis M--KPYYVTTAIAYPNAAPHVGHAY-EYIATDAIARFKRLDRYDVR---FLTGTDEHGLKVAQAAAAAGV-PTAA 68
B.melitensis MSREKYYITTAIAYPNGKPHIGHAY-ELIATDAMARFQRLNGMDVY---FLTGTDEHGIKMLQSARKEGI-TPRD 70
A.aeolicus TLMKKFYVTTPIYYVNDVPHLGHAY-TTIAADTIARYYRLRDYDVF---FLTGTDEHGLKIQKKAEELGI-SPKE 71
T.brucei KVEKVFFVTSPIYYVNAAPHIGHVY-STLITDVIGRYHRVKGERVF---ALTGTDEHGQKVAEAAKQKQV-SPYD 306
L.major KKQKVFFATTPIYYVNASPHIGHVY-STLIVDVLGRYHRVKGEEVF---VMTGTDEHGQKVAEAAAKQGV-SPMD 275
S.cerevisiae_m LYSKVSHVTTPIFYPNAKPHLGHLY-SSLLSDVYHRWQLFKGNLSF---FTTGTDEHGLKIQCASESNGFDQPKK 80
H.sapiens_m ACDVRAYFTTPIFYVNAAPHIGHLY-SALLADALCRHRRLRGPSTAATRFSTGTDEHGLKIQQAAATAGL-APTE 112
MetRS2
P.abyssi M--VRYMVTSALPYANGPIHAGHLAGAYLPADIFVRYLRLKGEDVV---FICGTDEHGTPISFRALKEGR-SPRE 69
E.coli -VAKKILVTCALPYANGSIHLGHML-EHIQADVWVRYQRMRGHEVN---FICADDAHGTPIMLKAQQLGI-TPEQ 71
K.pneumoniae -VAKKILVTCALPYANGSIHLGHML-EHIQADVWVRYQRMRGHEVN---FICADDAHGTPIMLKAQQLGI-TPEQ 72
X.citri M-TRTALVTTALPYANGPLHLGHLV-GYIQADIWVRARRLRGDKTW---FVCADDTHGTPIMLAAEKAGV-TPEA 69
B.circulans M---SIFIGGAWPYANGSLHLGHVA-ALLPGDILARYYRQKGEKVL---YVSGSDCNGTPISIRAQQENT-SVQE 67
P.aeruginosa -EPRKILVTSALPYANGSIHLGHML-EYIQTDMWVRFQKMRGNQAV---YVCADDAHGSAIMLRAEREGI-TSEQ 71
S.cerevisiae_c PNERNILITSALPYVNNVPHLGNIIGSVLSADIFARYCKGRNYNAL---FICGTDEYGTATETKALEEGV-TPRQ 263
H.sapiens_c AGERNVLITSALPYVNNVPHLGNIIGCVLSADVFARYSRLRQWNTL---YLCGTDEYGTATETKALEEGL-TPQE 331
AD
CP domain
α3 β3 α4 β4 β5 α5
80 90 100 110 120 130 140
Glu132
MetRS1
S.aureus YLDEMIAGIKQLWAKLEISNDDFIRTTEERHKHVVEQVFERLLKQGDIYLGEYEGWYSVPDETYYTESQL----- 140
T.thermophilus FVDRVSGRFKRAWDLLGIAYDDFIRTTEERHKKVVQLVLKKVYEAGDIYYGEYEGLYCVSCERFYTEKEL----- 139
M.tuberculosis LARRNSDVFQRMQEALNISFDRFIRTTDADHHEASKELWRRMSAAGDIYLDNYSGWYSVRDERFFVESETQL--- 140
B.melitensis LADRNTSAFRRMAEVLNSSNDDYIRTSEERHYKASQAIWQAMVANGDIYKGGYAGWYSVRDEAYYGEEETEV--- 142
A.aeolicus LVDRNAERFKKLWEFLKIEYTKFIRTTDPYHVKFVQKVFEECYKRGDIYLGEYEGWYCVGCEEFKSEAEL----- 141
T.brucei FTAAVAGEFKKCFEQMDYSIDYFIRTTNEQHKAVVKELWTKLEQKGDIYLGRYEGWYSISDESFLTPQNI----- 376
L.major FTTSVSSEFKQCFQEMNYDMNYFIRTTNPTHEKLVQDIWKKLAAKGDIYLGKYEGWYSVSDESFLTAQNV----- 345
S.cerevisiae_m FVDKLYPEFVQLDKIYGINYTRFIRTTDPDHIENVMKLWELCLKNGYIYMGEHKGWYSISDETFYPESKV----- 150
H.sapiens_m LCDRVSEQFQQLFQEAGISCTDFIRTTEARHRVAVQHFWGVLKSRGLLYKGVYEGWYCASDECFLPEAKV----- 182
knuckle 1
MetRS2
P.abyssi IVDEFHEQIKITFQRAKISFDFFGRTELPIHYKLSQEFFLKAYENGHLVKKVTKQAYCEHDKMFLPDRFVIGTCP 144
E.coli MIGEMSQEHQTDFAGFNISYDNYHSTHSEENRQLSELIYSRLKENGFIKNRTISQLYDPEKGMFLPDRFVKGTCP 146
K.pneumoniae MIGEMSQEHQTDFAGFDISYDNYHSTHSDENRELSELIYTRLKENGFIKNRTISQLYDPEKGMFLPDRFVKGTCP 147
X.citri FIANVQASHERDFAAFGVTFDHYDSTNSPVNRELTEAFYAKLEAAGHISRRSVAQFYDTAKGMFLPDRYIKGICP 144
B.circulans IADRYHAEFSQCFSKLGFSYDLFTRTDAKHHHASVQEIFLSLLQKGYLSKKTVEQAYCQKNNQFLPDRFVEGICP 142
P.aeruginosa LIDAVRAEHMGDFADFLVDFDNYHSTHSEENRELSSAIYLKLRDAGHIDTRPVTQYFDPEKQMFLADRFIKGTCP 146
S.cerevisiae_c LCDKYHKIHSDVYKWFQIGFDYFGRTTTDKQTEIAQHIFTKLNSNGYLEEQSMKQLYCPVHNSYLADRYVEGECP 338
H.sapiens_c ICDKYHIIHADIYRWFNISFDIFGRTTTPQQTKITQDIFQQLLKRGFVLQDTVEQLRCEHCARFLADRFVEGVCP 406
Arg399
β6 β7 β8 α6 α7
150 160 170 180 190
MetRS1
S.aureus -VDPQYENGKII-------------GGKSPDS-GHEVELVKEESYFFNISKYTDRLLEFYDQ-NPDFIQPPSRKN 199
T.thermophilus -VEG-----------------------LCPIH-GRPVERRKEGNYFFRMEKYRPWLQEYIQE-NPDLIRPEGYRN 188
M.tuberculosis -VDGT----------------------RLTVETGTPVTWTEEQTYFFRLSAYTDKLLAHYHA-NPDFIAPETRRN 191
B.melitensis RADGV----------------------RYGPQ-GTPVEWVEEESYFFRLSAYQDKLLDLYEN-NPGFIMPAERRN 193
A.aeolicus -AEDH----------------------TCPIH-QKKCEYIKEPSYFFRLSKYQDKLLELYEK-NPEFIQPDYRRN 191
T.brucei -TDGVDKDGNPC---------------KVSLESGHVVTWVSEENYMFRLSAFRERLLEWYHA-NPGCIVPEFRRR 434
L.major -ADGVDRDGKPC---------------KVSLESGHVVTWVEEENYMFRLSAFRERLLKYFHD-HPNCIVPEFRRR 403
S.cerevisiae_m -IKDPKNDGK-----------------YLNTESKNEVVYQSETNYFFRLSLFNKKIVDHIRK-NPDFIFPASKRD 206
H.sapiens_m -TQQPGPSGDSF---------------PVSLESGHPVSWTKEENYIFRLSQFRKPLQRWLRG-NPQAITPEPFHH 240
knuckle 2
knuckle 1
MetRS2
P.abyssi YCGAEDQKGDQCEVCGRPLTPEILINPRCAIC-GRPISFRDSAHYYIKMQDFAERLKRWIEK-QP---WKPNVKN 214
E.coli KCKSPDQYGDNCEVCGATYSPTELIEPKSVVS-GATPVMRDSEHFFFDLPSFSEMLQAWTRS----GALQEQVAN 216
K.pneumoniae KCKSPDQYGDNCEVCGATYSPTELIDPKSVVS-GATPVMRDSEHFFFDLPSFSEMLQAWTRS----GALQEQVAN 217
X.citri NCGSPDQYGDNCEVCGATYAPTELKEPKSVIS-GATPELRDSEHFFFEVGHFDGFLREWLAG----DVALPGVKA 214
B.circulans YCKQR-ARGDQCDHCSSILDPLELEDKRCKIC-GEEPVIKKTEHFYFAFSNFQKELENYVKQAEDNQLWNENAVS 215
P.aeruginosa KCGTADQYGDNCEACGATYAPTELKDPKSAIS-GATPVLKESLHYFFKLPDFEAMLKQWTRS----GALQESVAN 216
S.cerevisiae_c KCHYDDARGDQCDKCGALLDPFELINPRCKLD-DASPEPKYSDHIFLSLDKLESQISEWVEKASEEGNWSKNSKT 412
H.sapiens_c FCGYEEARGDQCDKCGKLINAVELKKPQCKVC-RSCPVVQSSQHLFLDLPKLEKRLEEWLGRTLPGSDWTPNAQF 480

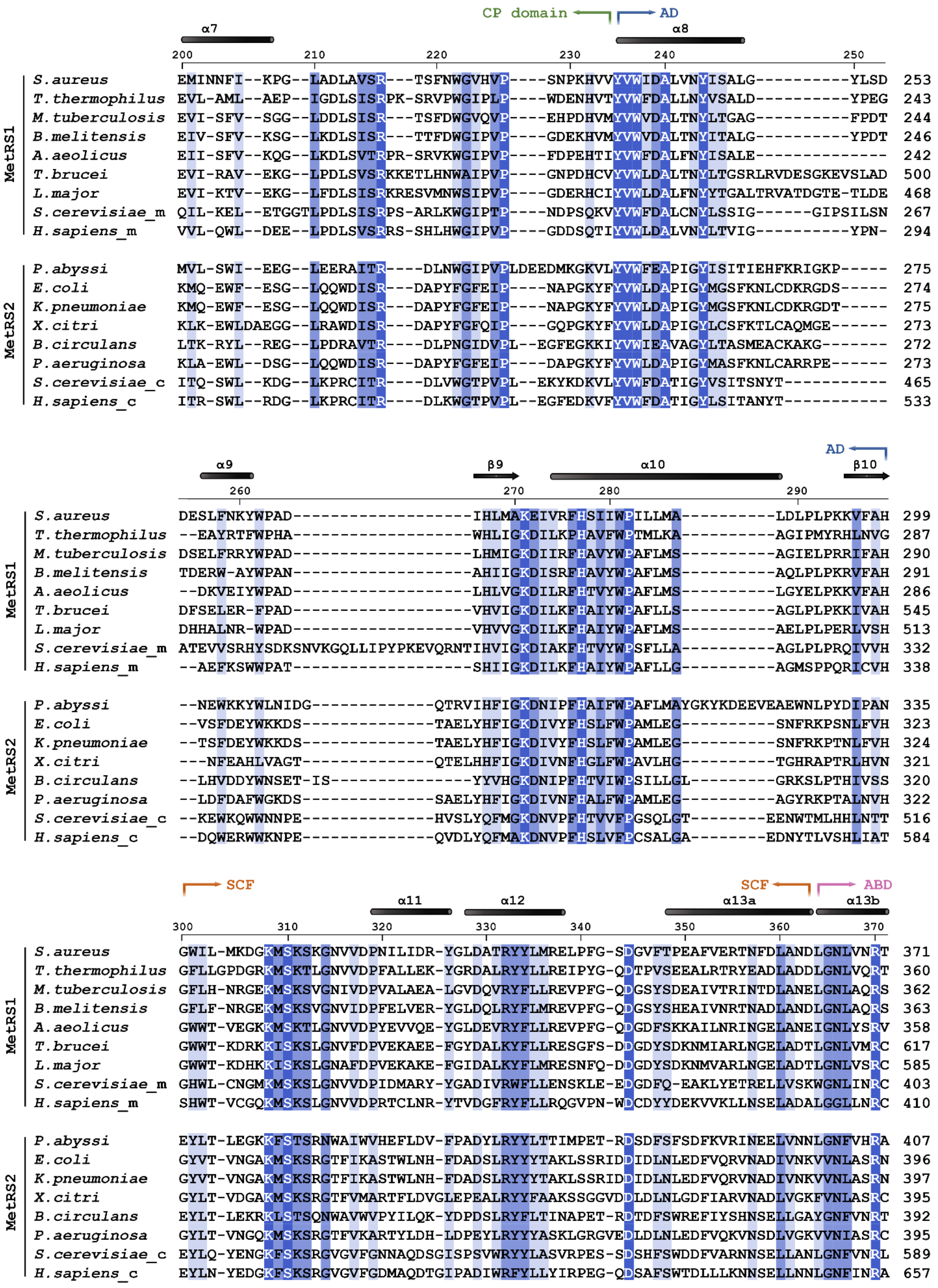
CP domain
AD
α7
α8
200 210 220 230 240 250
MetRS1
S.aureus EMINNFI--KPG--LADLAVSR---TSFNWGVHVP----SNPKHVVYVWIDALVNYISALG----------YLSD 253
T.thermophilus EVL-AML--AEP--IGDLSISRPK-SRVPWGIPLP----WDENHVTYVWFDALLNYVSALD----------YPEG 243
M.tuberculosis EVI-SFV--SGG--LDDLSISR---TSFDWGVQVP----EHPDHVMYVWVDALTNYLTGAG----------FPDT 244
B.melitensis EIV-SFV--KSG--LKDLSISR---TTFDWGIPVP----GDEKHVMYVWVDALTNYITALG----------YPDT 246
A.aeolicus EII-SFV--KQG--LKDLSVTRPR-SRVKWGIPVP----FDPEHTIYVWFDALFNYISALE-------------- 242
T.brucei EVI-RAV--EKG--LPDLSVSRKKETLHNWAIPVP----GNPDHCVYVWLDALTNYLTGSRLRVDESGKEVSLAD 500
L.major EVI-KTV--EKG--LFDLSISRKRESVMNWSIPVP----GDERHCIYVWLDALFNYYTGALTRVATDGTE-TLDE 468
S.cerevisiae_m QIL-KEL--ETGGTLPDLSISRPS-ARLKWGIPTP----NDPSQKVYVWFDALCNYLSSIG------GIPSILSN 267
H.sapiens_m VVL-QWL--DEE--LPDLSVSRRS-SHLHWGIPVP----GDDSQTIYVWLDALVNYLTVIG----------YPN- 294
MetRS2
P.abyssi MVL-SWI--EEG--LEERAITR----DLNWGIPVPLDEEDMKGKVLYVWFEAPIGYISITIEHFKRIGKP----- 275
E.coli KMQ-EWF--ESG--LQQWDISR---DAPYFGFEIP----NAPGKYFYVWLDAPIGYMGSFKNLCDKRGDS----- 274
K.pneumoniae KMQ-EWF--ESG--LQQWDISR---DAPYFGFEIP----NAPGKYFYVWLDAPIGYMGSFKNLCDKRGDT----- 275
X.citri KLK-EWLDAEGG--LRAWDISR---DAPYFGFQIP----GQPGKYFYVWLDAPIGYLCSFKTLCAQMGE------ 273
B.circulans LTK-RYL--REG--LPDRAVTR----DLPNGIDVPL--EGFEGKKIYVWIEAVAGYLTASMEACKAKG------- 272
P.aeruginosa KLA-EWL--DSG--LQQWDISR---DAPYFGFEIP----DAPGKYFYVWLDAPIGYMASFKNLCARRPE------ 273
S.cerevisiae_c ITQ-SWL--KDG--LKPRCITR----DLVWGTPVPL--EKYKDKVLYVWFDATIGYVSITSNYT----------- 465
H.sapiens_c ITR-SWL--RDG--LKPRCITR----DLKWGTPVPL--EGFEDKVFYVWFDATIGYLSITANYT----------- 533
AD
α9
β9
α10
β10
260 270 280 290
MetRS1
S.aureus DESLFNKYWPAD------------------IHLMAKEIVRFHSIIWPILLMA----------LDLPLPKKVFAH 299
T.thermophilus --EAYRTFWPHA------------------WHLIGKDILKPHAVFWPTMLKA----------AGIPMYRHLNVG 287
M.tuberculosis DSELFRRYWPAD------------------LHMIGKDIIRFHAVYWPAFLMS----------AGIELPRRIFAH 290
B.melitensis TDERW-AYWPAN------------------AHIIGKDISRFHAVYWPAFLMS----------AQLPLPKRVFAH 291
A.aeolicus --DKVEIYWPAD------------------LHLVGKDILRFHTVYWPAFLMS----------LGYELPKKVFAH 286
T.brucei DFSELER-FPAD------------------VHVIGKDILKFHAIYWPAFLLS----------AGLPLPKKIVAH 545
L.major DHHALNR-WPAD------------------VHVVGKDILKFHAIYWPAFLMS----------AELPLPERLVSH 513
S.cerevisiae_m ATEVVSRHYSDKSNVKGQLLIPYPKEVQRNTIHVIGKDIAKFHTVYWPSFLLA----------AGLPLPRQIVVH 332
H.sapiens_m --AEFKSWWPAT------------------SHIIGKDILKFHAIYWPAFLLG----------AGMSPPQRICVH 338
MetRS2
P.abyssi --NEWKKYWLNIDG-------------QTRVIHFIGKDNIPFHAIFWPAFLMAYGKYKDEEVEAEWNLPYDIPAN 335
E.coli --VSFDEYWKKDS--------------TAELYHFIGKDIVYFHSLFWPAMLEG----------SNFRKPSNLFVH 323
K.pneumoniae --TSFDEYWKKDS--------------TAELYHFIGKDIVYFHSLFWPAMLEG----------SNFRKPTNLFVH 324
X.citri ---NFEAHLVAGT--------------QTELHHFIGKDIVNFHGLFWPAVLHG----------TGHRAPTRLHVN 321
B.circulans --LHVDDYWNSET-IS---------------YYVHGKDNIPFHTVIWPSILLGL---------GRKSLPTHIVSS 320
P.aeruginosa --LDFDAFWGKDS--------------SAELYHFIGKDIVNFHALFWPAMLEG----------AGYRKPTALNVH 322
S.cerevisiae_c --KEWKQWWNNPE--------------HVSLYQFMGKDNVPFHTVVFPGSQLGT--------EENWTMLHHLNTT 516
H.sapiens_c --DQWERWWKNPE--------------QVDLYQFMAKDNVPFHSLVFPCSALGA--------EDNYTLVSHLIAT 584
SCF
SCF
ABD
α11
α12
α13a
α13b
300 310 320 330 340 350 360 370
MetRS1
S.aureus GWIL-MKDGKMSKSKGNVVDPNILIDR-YGLDATRYYLMRELPFG-SDGVFTPEAFVERTNFDLANDLGNLVNRT 371
T.thermophilus GFLLGPDGRKMSKTLGNVVDPFALLEK-YGRDALRYYLLREIPYG-QDTPVSEEALRTRYEADLADDLGNLVQRT 360
M.tuberculosis GFLH-NRGEKMSKSVGNIVDPVALAEA-LGVDQVRYFLLREVPFG-QDGSYSDEAIVTRINTDLANELGNLAQRS 362
B.melitensis GFLF-NRGEKMSKSVGNVIDPFELVER-YGLDQLRYFLMREVPFG-QDGSYSHEAIVNRTNADLANDLGNLAQRS 363
A.aeolicus GWWT-VEGKKMSKTLGNVVDPYEVVQE-YGLDEVRYFLLREVPFG-QDGDFSKKAILNRINGELANEIGNLYSRV 358
T.brucei GWWT-KDRKKMSKSLGNVFDPVEKAEE-FGYDALKYFLLRESGFS-DDGDYSDKNMIARLNGELADTLGNLVMRC 617
L.major GWWT-KDHKKISKSLGNAFDPVEKAKE-FGIDALKYFLMRESNFQ-DDGDYSDKNMVARLNGELADTLGNLVSRC 585
S.cerevisiae_m GHWL-CNGMKMSKSLGNVVDPIDMARY-YGADIVRWFLLENSKLE-EDGDFQ-EAKLYETRELLVSKWGNLINRC 403
H.sapiens_m SHWT-VCGQKMSKSLGNVVDPRTCLNR-YTVDGFRYFLLRQGVPN-WDCDYYDEKVVKLLNSELADALGGLLNRC 410
MetRS2
P.abyssi EYLT-LEGKKFSTSRNWAIWVHEFLDV-FPADYLRYYLTTIMPET-RDSDFSFSDFKVRINEELVNNLGNFVHRA 407
E.coli GYVT-VNGAKMSKSRGTFIKASTWLNH-FDADSLRYYYTAKLSSRIDDIDLNLEDFVQRVNADIVNKVVNLASRN 396
K.pneumoniae GYVT-VNGAKMSKSRGTFIKASTWLNH-FDADSLRYYYTAKLSSRIDDIDLNLEDFVQRVNADIVNKVVNLASRN 397
X.citri GYLT-VDGAKMSKSRGTFVMARTFLDVGLEPEALRYYFAAKSSGGVDDLDLNLGDFIARVNADLVGKFVNLASRC 395
B.circulans EYLT-LEKRKLSTSQNWAVWVPYILQK-YDPDSLRYFLTINAPET-RDTDFSWREFIYSHNSELLGAYGNFVNRT 392
P.aeruginosa GYLT-VNGQKMSKSRGTFVKARTYLDH-LDPEYLRYYYASKLGRGVEDLDLNLEDFVQKVNSDLVGKVVNIASRC 395
S.cerevisiae_c EYLQ-YENGKFSKSRGVGVFGNNAQDSGISPSVWRYYLASVRPES-SDSHFSWDDFVARNNSELLANLGNFVNRL 589
H.sapiens_c EYLN-YEDGKFSKSRGVGVFGDMAQDTGIPADIWRFYLLYIRPEG-QDSAFSWTDLLLKNNSELLNNLGNFINRA 657

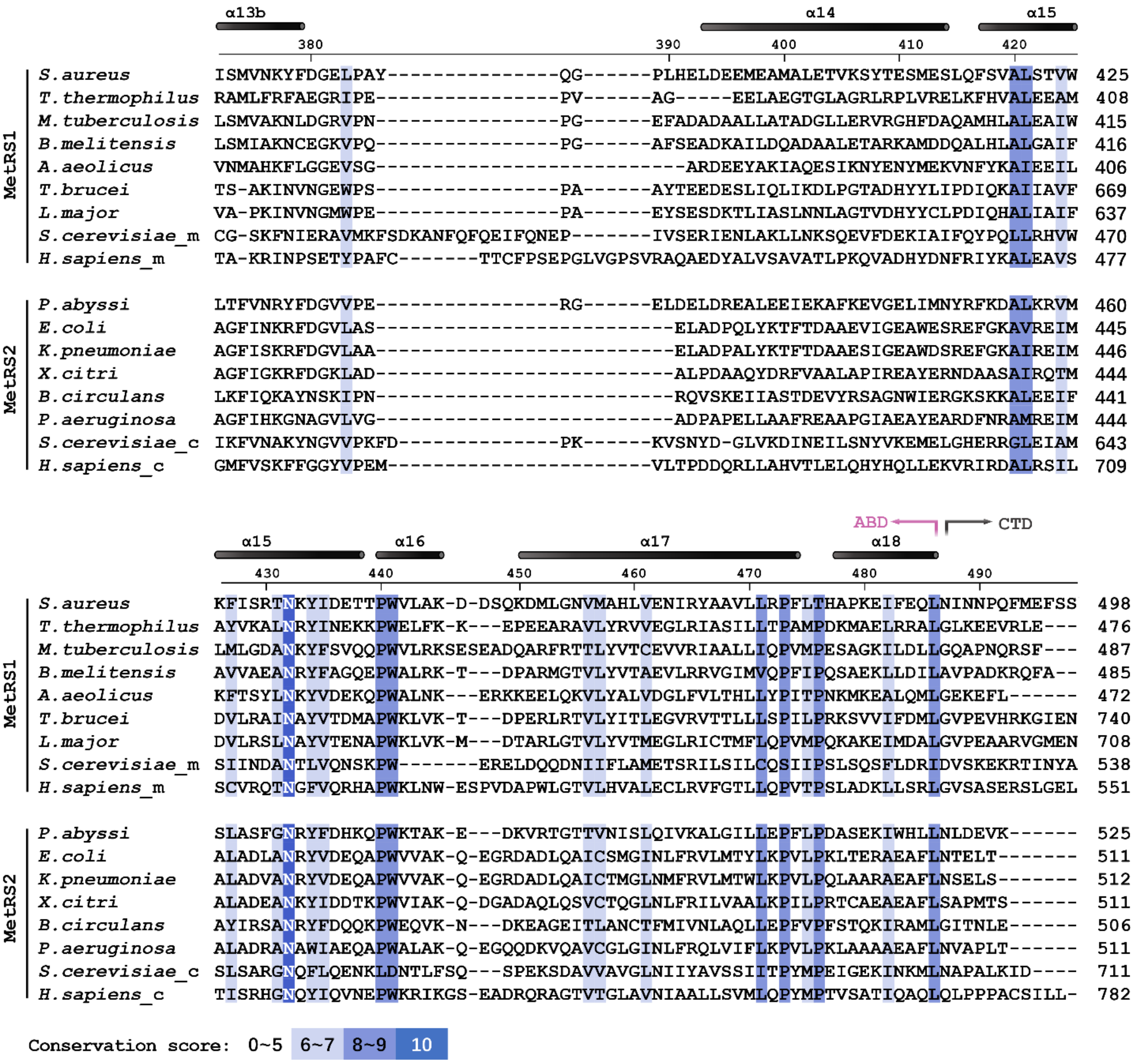

Supplementary Figure S2. Sequence alignments of MetRS proteins from different organisms. The sequences of MetRS1 proteins from *Staphylococcus aureus* (UniProtKB ID: A0A0D6FXK4), *Aquifex aeolicus* (UniProtKB ID: O67298), *Thermus thermophilus* (UniProtKB ID: P23395), *Mycobacterium tuberculosis* (UniProtKB ID: P9WFU5), *Brucella melitensis* (UniProtKB ID: Q2YQ76), *Trypanosoma brucei* (UniProtKB ID: Q38C91), *Leishmania major* (UniProtKB ID: Q4QCD2), *Saccharomyces cerevisiae* mitochondria (UniProtKB ID: P22438), and *Homo sapiens* mitochondria (UniProtKB ID: Q96GW9) and MetRS2 proteins from *Pyrococcus abyssi* (UniProtKB ID: Q9V011), *Bacillus circulans* (UniProtKB ID: A0A553SHI8), *Klebsiella pneumoniae* (UniProtKB ID: W9BHW1), *Pseudomonas aeruginosa* (UniProtKB ID: A0A3M5D7V4), *Escherichia coli* (UniProtKB ID: P00959), *Saccharomyces cerevisiae* cytoplasm (UniProtKB ID: P00958) and *Homo sapiens* cytoplasm (UniProtKB ID: P56192) were aligned using MAFFT program (64) with manual adjustments. The secondary structures corresponding to *Sa*MetRS are shown above the sequences. Knuckles 1 and 2 in the CP domain are shown

in red and green boxes, respectively. The residues Lys56 and Glu132 in *Sa*MetRS as well as Arg399 in *Hc*MetRS discussed in the paper are presented as red text.

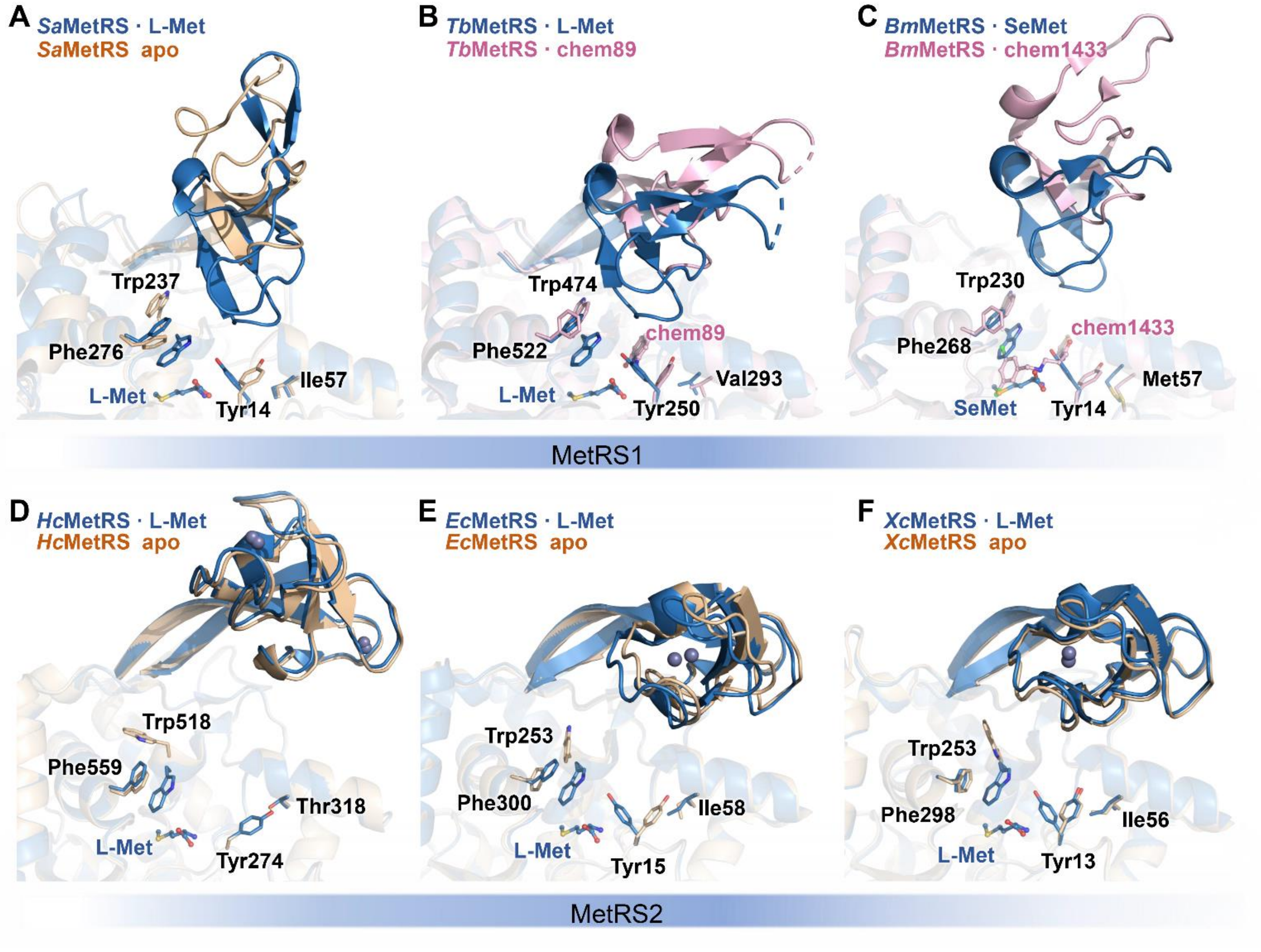

Supplementary Figure S3. The conformation of the CP domain in MetRS1 and MetRS2 upon L-Met binding. (A-C) The knuckle structure in the CP domain of MetRS1, including *Sa*MetRS **(A)**, *Tb*MetRS **(B)** and *Bm*MetRS **(C)**, bent inward at the M-state (blue) compared to that of the apo (wheat) or inhibitor-bound (pink) states. **(D-F)** L-Met binding caused few conformational changes in the CP domain in the structures of MetRS2 proteins, including *Hc*MetRS **(D)**, *Ec*MetRS **(E)** and *Xc*MetRS **(F)**. The structures used for comparison are *Tb*MetRS·L-Met (PDB ID: 4EG1), *Tb*MetRS·chem89 (PDB ID: 4EG8), *Bm*MetRS·selenomethionine (PDB ID: 4DLP), *Bm*MetRS·chem1433 (PDB ID: 4PY2), *Hc*MetRS·L-Met (PDB ID: 5GOY), *Hc*MetRS apo (PDB ID: 5GL7), *Ec*MetRS·L-Met (PDB ID: 1F4L), *Ec*MetRS apo (PDB ID: 1QQT), *Xc*MetRS·L-Met (PDB ID: 6WQ6), *Xc*MetRS apo (PDB ID: 6WQI).

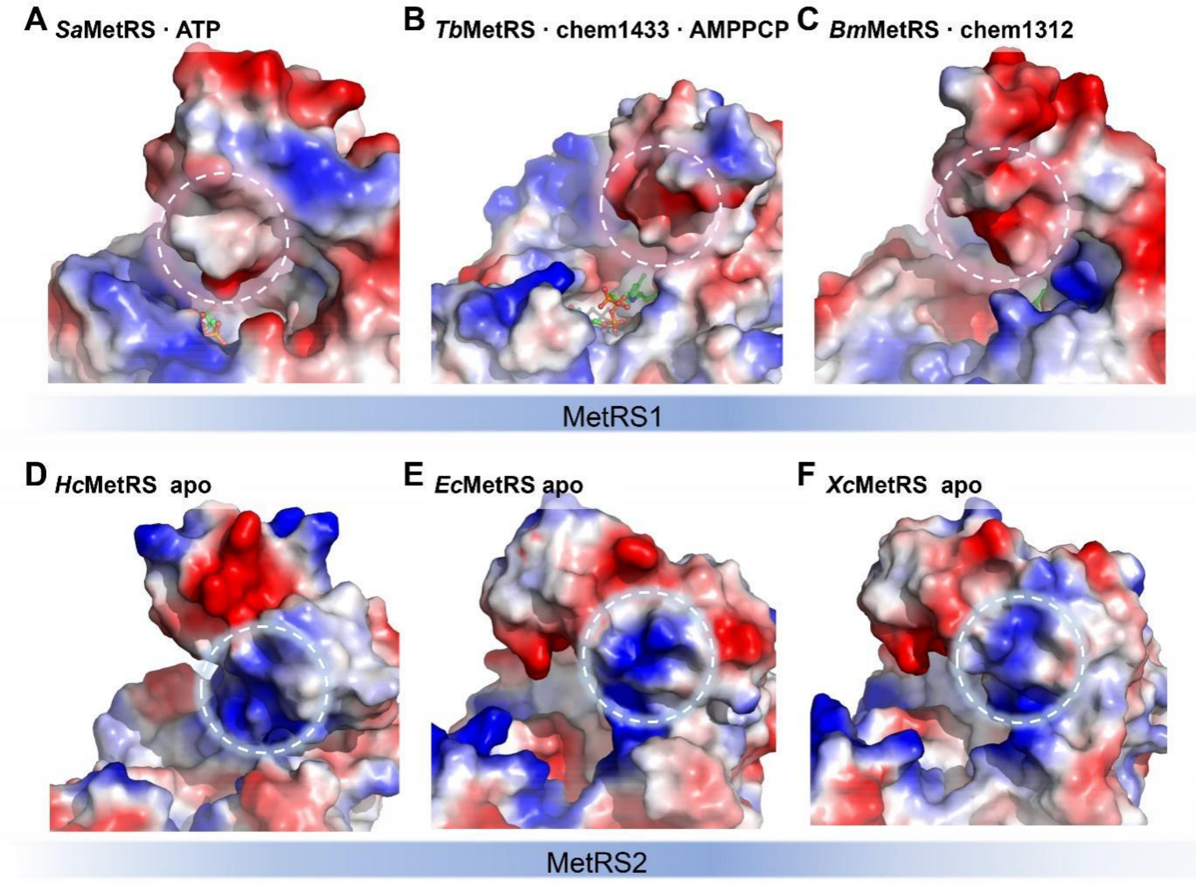

Supplementary Figure S4. Electrostatics analyses of CP knuckles in MetRS1 and MetRS2 proteins. **(A-C)** The CP knuckle surface facing the ATP site is mainly negatively charged in MetRS1. **(D-F)** The CP knuckle surface facing the ATP site is mainly positively charged in MetRS2. The structures used for analysis were *Tb*MetRS·chem1433·AMPPCP (PDB ID: 4MWD), *Bm*MetRS·chem1312 (PDB ID: 5K0S), *Hc*MetRS apo (PDB ID: 5GL7), *Ec*MetRS apo (PDB ID: 1QQT), and *Xc*MetRS apo (PDB ID: 6WQI).

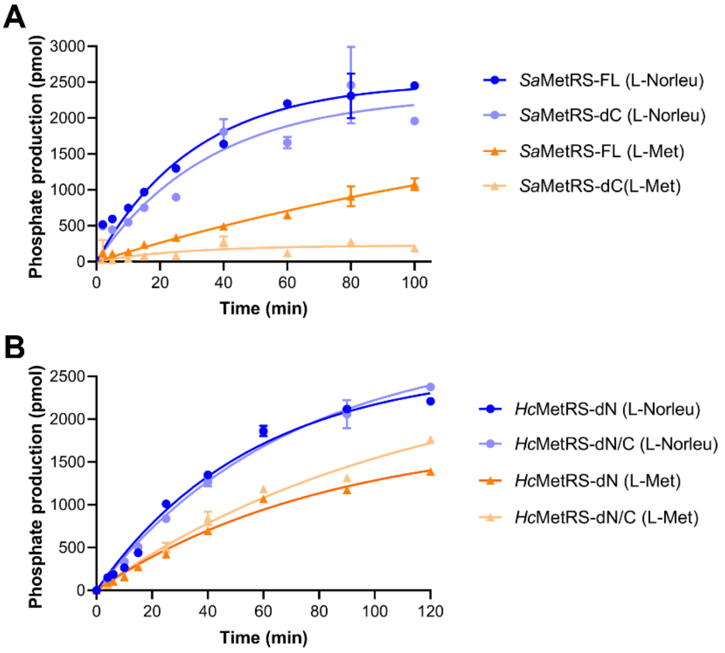

Supplementary Figure S5. Pretransfer editing activity-based enzyme assay for MetRS. (A) The pretransfer editing activities of full-length *Sa*MetRS (*Sa*MetRS-FL) and C-terminal truncated *Sa*MetRS (*Sa*MetRS-dC) in the presence of L-norleucine (L-Norleu) or L-methionine (L-Met) were evaluated by measuring phosphate production. **(B)** The pretransfer editing activities of N-terminal part truncated *Hc*MetRS (*Hc*MetRS-dN) and both N- and C-terminal parts truncated *Hc*MetRS (*Hc*MetRS-dN/C) in the presence of L-Norleu or L-Met were evaluated by measuring phosphate production. The tRNA-independent pretransfer editing was much more efficient for noncognate norleucyl adenylate than for methionyl adenylate, resulting in a higher signal for activity measurement. *Sa*MetRS-dC had a similar activity to *Sa*MetRS-FL in the transfer editing assay, supporting that we can use *Sa*MetRS-dC instead of *Sa*MetRS-FL in crystal structure analyses. However, for unknown reasons, low phosphate production was observed for *Sa*MetRS-dC in the presence of L-Met.

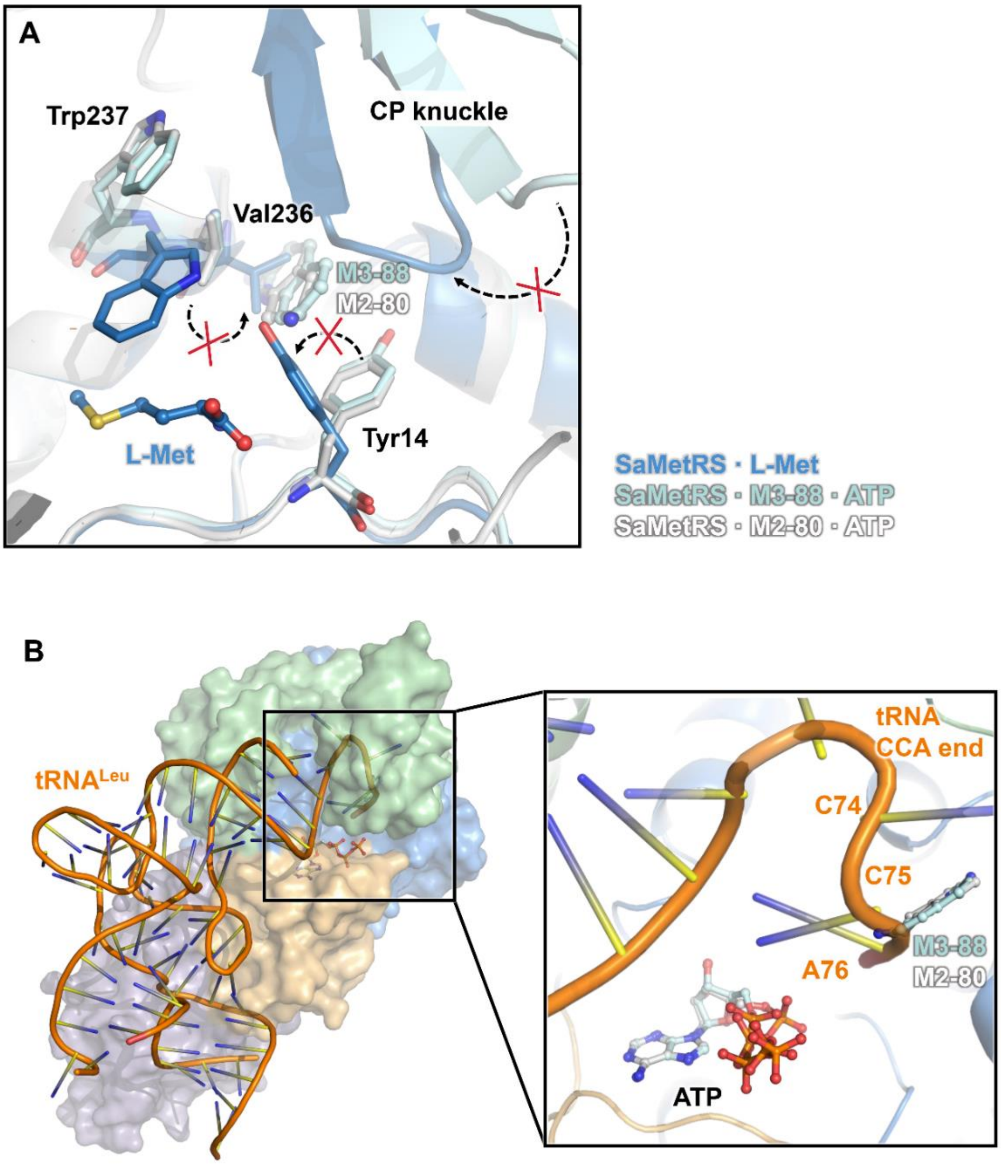

Supplementary Figure S6. Auxiliary site fragments inhibit the functional binding of substrates L-Met and tRNA^{Met} to *Sa*MetRS through an allosteric or an orthosteric manner, respectively. (A) Auxiliary site fragments blocked the conformational changes required by L-Met binding. Superimposition of the structures of the *Sa*MetRS·M3-88·ATP (palecyan) and *Sa*MetRS·M2-80·ATP (white) complexes with that of the *Sa*MetRS·L-Met complex (blue) revealed that the binding of fragments M3-88 and M2-80 to the auxiliary pocket both blocked the conformational changes of Tyr14 and Trp237, which are necessary for L-Met binding. **(B)** Auxiliary site fragments interfere with the functional binding of the CCA end of tRNA^{Met}. Superimposition of the structures of *Sa*MeRS·M3-88·ATP and *Sa*MeRS·M2-80·ATP with that of the *Ec*LeuRS·tRNA^{Leu} complex (PDB ID: 4AQ7) revealed that fragments M3-88 (palecyan) and M2-80 (white) partially overlapped with nucleotide A76 of tRNA^{Met}, which prevented the CCA end of tRNA^{Met} from accepting L-Met.

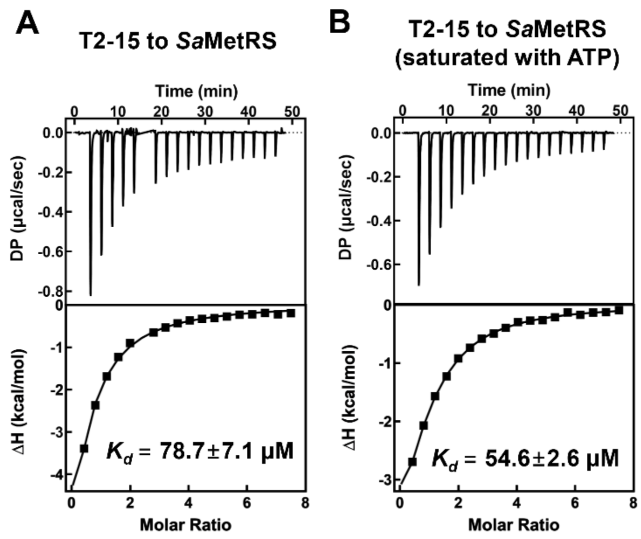

Supplementary Figure S7. ITC titrations of fragment T2-15 to *Sa*MetRS. ITC titrations of fragment T2-15 to *Sa*MetRS alone **(A)** and saturated with ATP **(B)** presented similar binding affinities.

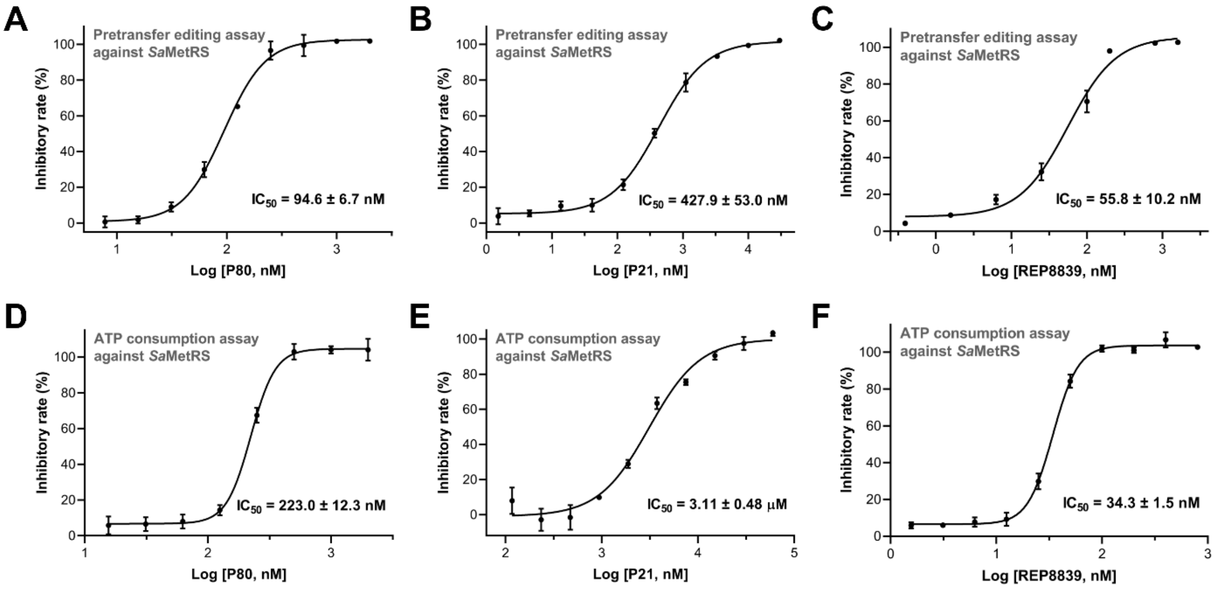

Supplementary Figure S8. The inhibitory activity of compounds P80 and P21 for *Sa*MetRS. (A-B) The inhibition of compounds P80 and P21 against *Sa*MetRS measured by the pretransfer editing assay. **(D-E)** The inhibition of compounds P80 and P21 against tRNA aminoacylation activity of *Sa*MetRS measured by the tRNA-dependent ATP consumption assay. **(C, F)** The reported inhibitor REP8839 was used as a positive control in the two assays.

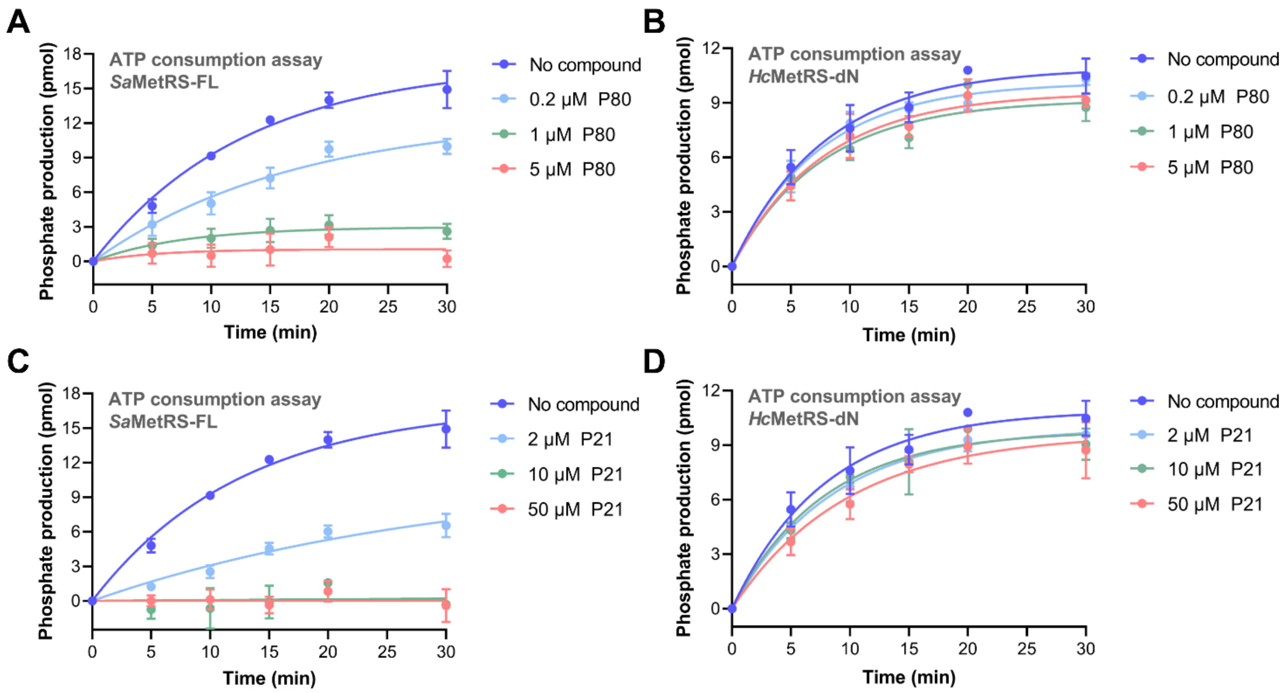

Supplementary Figure S9. Compounds P80 and P21 selectively inhibited *Sa*MetRS but not *Hc*MetRS in the ATP consumption assays. (A) P80 exhibited dose-dependent inhibition against *Sa*MetRS-FL. **(B)** P80 did not significantly inhibit *Hc*MetRS-dN even at high concentrations. **(C)** P21 exhibited dose-dependent inhibition against *Sa*MetRS-FL. **(D)** P21 did not significantly inhibit *Hc*MetRS even at high concentrations.

Supplementary table

Supplementary Table S1. Data collection and refinement statistics.

	*Sa*MetRS	*Sa*MetRS · L-Met	*Sa*MetRS · ATP	*Sa*MetRS · M3-146 · ATP	*Sa*MetRS · B54 · ATP
**Data collection**					
Wavelength (Å)	1.5418	1.5418	1.5418	0.9791	0.9791
Resolution (Å)	59.73-2.65(2.74-2.65)	62.10-2.80(2.90-2.80)	59.74-2.50(2.59-2.50)	61.45-2.40(2.49-2.40)	46.02-2.09(2.15-2.09)
Space group	$P2_12_12_1$	$P2_12_12_1$	$P2_12_12_1$	$P2_12_12_1$	$P2_12_12_1$
Cell dimensions					
a, *b*, *c* (Å)	59.7, 69.0, 117.4	59.3, 72.7, 119.0	59.7, 69.3, 118.0	60.9, 71.4, 120.8	59.3, 72.7, 118.7
α, β, γ (°)	90.0, 90.0, 90.0	90.0, 90.0, 90.0	90.0, 90.0, 90.0	90.0, 90.0, 90.0	90.0, 90.0, 89.8
Unique reflections	14046 (1402)	13216 (1327)	17489 (1715)	21257(2174)	30973(2306)
R_{merge}^{b}	0.03 (0.07)	0.05 (0.13)	0.07 (0.21)	0.11(0.56)	0.14 (0.47)
Average $I/\sigma(I)$	17.8 (8.7)	17.3 (7.0)	10.2 (3.6)	13.1(4.6)	9.4(4.1)
Completeness (%)	96.0 (97.0)	99.6 (100.0)	99.9 (99.7)	99.9(100.0)	99.7(97.0)
Redundancy	3.6 (3.7)	3.8 (4.0)	4.0 (4.1)	6.0(5.2)	6.5(6.0)
**Refinement**					
Resolution (Å)	59.48-2.65	62.09-2.80	59.73-2.50	61.45-2.40	46.02-2.09
No. reflections	13341	12506	16537	20145	29369
$R_{work}^{c}/R_{free}^{d}$ (%)	23.8/27.6	22.9 (27.9)	22.4/27.1	21.3(23.6)	21.3(25.0)
No. non-hydrogen atoms					
Protein	3955	4160	4059	4142	4192
Ligand/ion	0	9	39	44	57
Water oxygen atoms	5	12	32	23	85
Mean B factor (Å^2)	20.6	19.2	16.4	35.1	19.8
RMSD bond (Å)	0.007	0.007	0.008	0.007	0.002
RMSD angle (°)	1.13	1.12	1.30	1.15	1.18
Ramachandran plot (%)					
Favored	96.3	96.5	97.8	97.3	98.5
Allowed	3.5	3.3	2.2	2.7	1.5
Outliers	0.2	0.2	0.0	0.0	0.0

	*Sa*MetRS · M3-88 · ATP	*Sa*MetRS · M2-80 · ATP	*Sa*MetRS · P80	*Sa*MetRS · P21 · ATP
**Data collection**				
Wavelength (Å)	1.5418	0.9791	0.9791	1.5418
Resolution (Å)	62.40-2.80(2.95-2.80)	64.31-2.26(2.40-2.26)	66.40-1.92(2.03-1.92)	61.13-2.40(2.49-2.40)
Space group	$P2_12_12_1$	$P2_12_12_1$	$P2_12_12_1$	$P2_12_12_1$
Cell dimensions				
a, *b*, *c* (Å)	70.0, 73.6, 117.5	61.0, 76.5, 118.6	64.5, 80.3, 118.0	60.5, 72.1, 115.5
α, *β*, *γ* (°)	90.0, 90.0, 90.0	90.0, 90.0, 90.0	90.0, 90.0, 90.0	90.0, 90.0, 90.0
Unique reflections	15515 (2235)	24773 (4141)	44015(5951)	20367(2082)
R_{merge}[b]	0.16 (0.45)	0.07 (0.54)	0.07(0.65)	0.14(0.54)
Average $I/\sigma(I)$	5.6 (2.2)	12.2 (3.0)	15.8(3.2)	10.6(3.1)
Completeness (%)	99.8 (100.0)	93.2 (98.1)	93.0(87.4)	99.9(99.8)
Redundancy	4.2 (4.4)	3.4 (3.6)	5.9(5.5)	4.7(3.3)
**Refinement**				
Resolution (Å)	62.40-2.80	64.31-2.26	66.40-1.92	61.13-2.40
No. reflections	14630	23442	41654	19264
R_{work}[c]/R_{free}[d] (%)	26.2 (27.4)	22.0/26.6	21.1(23.7)	21.7(25.9)
No. non-hydrogen atoms				
Protein	4205	3903	4163	3859
Ligand/ion	46	63	60	83
Water oxygen atoms	13	35	68	28
Mean B factor ($Å^2$)	32.7	45.5	33.8	21.9
RMSD bond (Å)	0.008	0.003	0.004	0.007
RMSD angle (°)	1.24	1.21	1.21	1.22
Ramachandran plot (%)				
Favored	95.5	95.4	97.3	98.1
Allowed	4.3	4.6	2.7	1.9
Outliers	0.2	0.0	0.0	0.0

[a] Values in parentheses are for the highest resolution shell.

[b] $R_{merge} = \sum_h \sum_l |I(h)_l - \langle I(h)\rangle| / \sum_h \sum_l I(h)_l$, where $I(h)_l$ is the lth observation of the reflection h and $\langle I(h)\rangle$ is the weighted average intensity for all observations l of reflection h.

[c] $R_{work} = \sum h||F_{obs}(h)| - |F_{cal}(h)||/\sum_h|F_{obs}(h)|$, where $F_{obs}(h)$ and $F_{cal}(h)$ are the observed and calculated structure factors for reflection h respectively.

[d] R_{free} was calculate as R_{work} using 5% of the reflections which were selected randomly and omitted from refinement.

Synthetic Chemistry

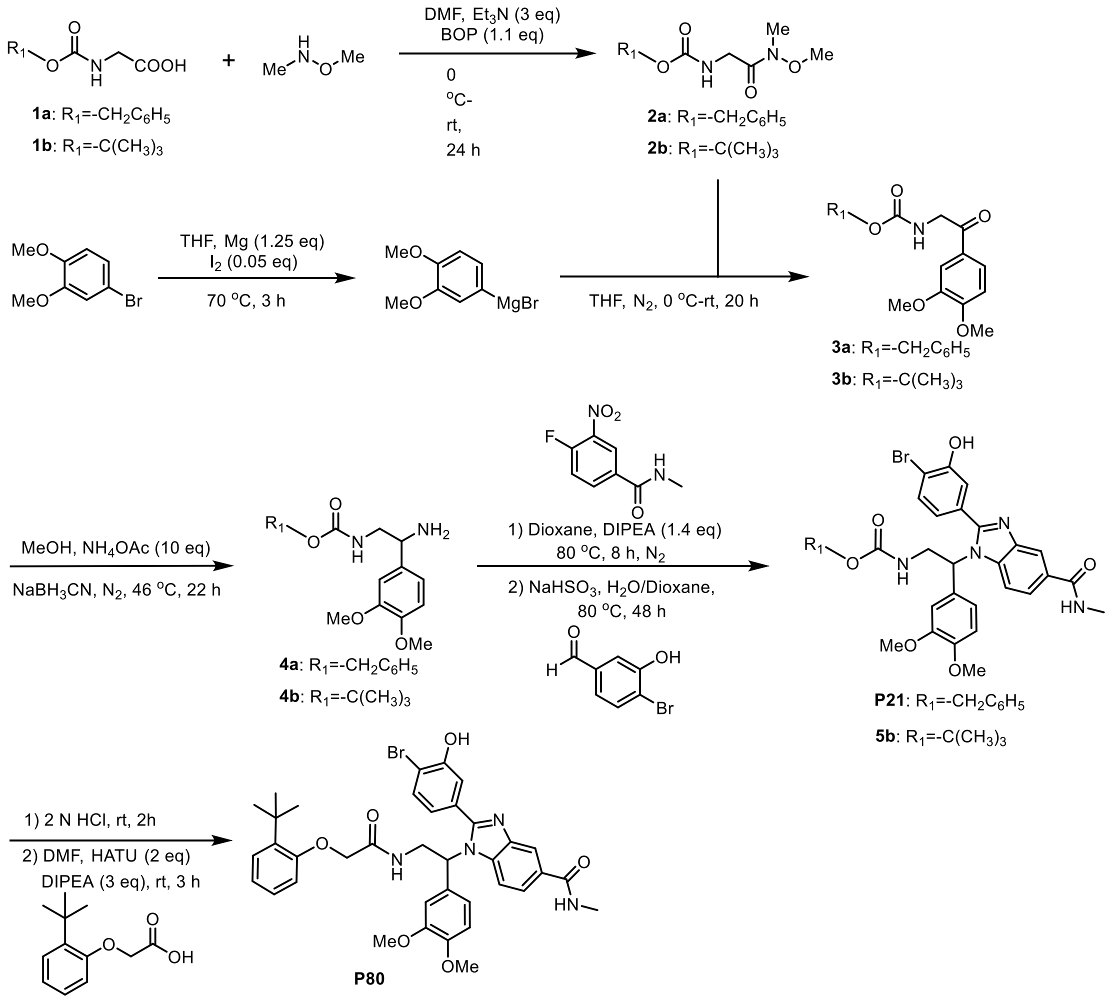

Supplementary Scheme S1. The synthesis of compound P21 and P80.

Benzyl (2-(methoxy(methyl)amino)-2-oxoethyl)carbamate (**2a**). Weinreb **2a** was synthesized as described previously (65) . A flask was added 2-(((benzyloxy)carbonyl)amino)acetic acid **1a** (6.25 g, 30 mmol, 1 equiv) and anhydrous DMF (60 mL), and the flask was cooled to 0 °C. Triethylamine (12.8 mL, 90 mmol), benzotriazol-1-yloxy-tris (dimethylamino)phosphonium (BOP) hexafluorophosphate (14.6 g, 33 mmol, 1.1 equiv) and N, O-dimethylhydroxyl amine hydrochloride (4.4 g, 45 mmol, 1.5 equiv) were added in sequence. The mixture was stirred at rt for 24 h. After completed, DCM (80 mL) was added, and the organic layer was washed with 1 N HCl and water (3 x 100 mL). The organic layer was dried with Na_2SO_4, filtered through celite, concentrated in vacuo, and purified through flash chromatography to obtain **2a** as a white solid (5.2 g, yield 82%). ^{1}H NMR (400 MHz, $CDCl_3$) δ 7.36 (dd, J = 17.9, 4.9 Hz, 5H), 5.14 (s, 2H), 4.16 (s, 2H), 3.73 (s, 3H), 3.22 (s, 3H).

Tert-butyl (2-(methoxy(methyl)amino)-2-oxoethyl)carbamate (**2b**). Compound **2b** was synthesis from **1b**. Light yellow oil, yield 75%. ^{1}H NMR (400 MHz, $CDCl_3$) δ 4.08 (d, J = 3.6 Hz, 2H), 3.72 (s, 3H), 3.21 (s, 3H), 1.46 (s, 9H).

Benzyl (2-amino-2-(3,4-dimethoxyphenyl)ethyl)carbamate (**4a**). Mg (345.6 mg, 14.4 mmol, 3.9 equiv), I_2 (62.9 mg, 0.24 mmol, 0.03 equiv) and anhydrous THF (15 mL) were added to a dry 100 mL round-bottom flask. 4-bromo-1,2-dimethoxybenzene (2.6 g, 10 mmol, 3.0 equiv) in anhydrous THF was added dropwise to the flask at 70 °C over 10 min. The reaction was stirred at 70 °C for another 1 h to get Grignard reagent. The Grignard reagent was cooled to rt, and was added dropwise to the mixture of **2a** (1.0 g, 4.0 mmol, 1 equiv) in anhydrous THF at 0 °C. Then, the mixture was stirred at rt for 10 h under N_2 atmosphere. After finished, the solvent was evaporated, and the residue was redissolved with EtOAc, washed with H_2O, saturated $NaHCO_3$ and NaCl. The organic layer was dried, evaporated to afford crude product **3a**, which was used directly for the next step.

3a and NH_4OAc (1.7 g, 22.5 mmol, 10 equiv) were dissolved with anhydrous MeOH (20 mL). Then, $NaBH_3CN$ (253 mg, 4.1 mmol, 1.8 equiv) in anhydrous MeOH (6 mL) was added to the mixture through syringe. The flask was stirred at 45 °C for 24 h under N_2 atmosphere. The reaction was stopped, and the solvent was removed. Next, the residue was redissolved with DCM, H_2O and 1 N NaOH aqueous solution. The organic layer was separated and washed with NaCl aqueous solution, dried over anhydrous Na_2SO_4, filtered and evaporated. The crude residue was purified with flash chromatography to get **4a** (362.3 mg, yield 27.4%) as a white solid. ^{1}H NMR (400 MHz, $CDCl_3$) δ 7.30–7.13 (m, 5H), 6.81–6.62 (m, 3H), 5.37 (s, 1H), 5.02–4.95 (m, 2H), 3.92 (t, J = 6.3 Hz, 1H), 3.74 (s, 6H), 3.29–3.25 (m, 1H), 3.22–3.11 (m, 1H).

Tert-butyl (2-amino-2-(3,4-dimethoxyphenyl)ethyl)carbamate (**4b**). **4b** was synthesis the same as **4a**. Light yellow oil, yield 40%. ^{1}H NMR (400 MHz, $CDCl_3$) δ 6.81 (d, J = 4.5 Hz, 1H), 6.77 (dd, J = 11.0, 4.9 Hz, 2H), 4.97 (t, J = 5.7 Hz, 1H), 3.94 (dd, J = 7.4, 5.8 Hz, 1H), 3.80 (s, 3H), 3.78 (s, 3H), 3.28–3.20 (m, 1H), 3.14 (dd, J = 12.9, 6.4 Hz, 1H), 1.35 (s, 9H).

Benzyl(2-(2-(4-bromo-3-hydroxyphenyl)-5-(methylcarbamoyl)-1H-benzo[d]imidazol-1-yl)-2-(3,4-dimethoxyphenyl)ethyl)carbamate (**P21**). **P21** was synthesized according to the literature (56). To a flask was added **4a** (235 mg, 0.71 mmol, 1 equiv), 4-fluoro-N-methyl-3-nitrobenzamide (141 mg, 0.71 mmol, 1 equiv), N,N-diisopropylethylamine (DIPEA) (0.173 mL, 0.99 mmol) and 1,4-dioxane (10 mL) at room temperature. The reaction was heated at 80 °C for 8 h under N_2 atmosphere. Then, sodium dithionite (372 mg, 2.14 mmol), 4-bromo-3-hydroxybenzaldehyde (143 mg, 0.71 mmol) and water (0.75 mL) was added. The reaction was heated at 80 °C for another 48 h. The reaction was concentrated under vacuum and the residue was purified with flash chromatography to give **P21** (215 mg, 46% yield) as a white solid. ^{1}H NMR (400 MHz, CD_3OD) δ 8.18 (d, J = 10.4 Hz, 1H), 7.65 (dd, J = 21.2, 8.4 Hz, 1H), 7.52 (d, J = 7.8 Hz, 1H), 7.37–7.19 (m, 6H), 7.12 (s, 1H), 6.98–6.83 (m, 4H), 5.99–5.91 (m, 1H), 4.14 (dd, J = 14.2, 4.6 Hz, 1H), 4.02–3.90 (m, 1H), 3.82 (s, 3H), 3.71 (s, 3H), 2.94 (s, 3H). ^{13}C NMR (101 MHz, CD_3OD) δ 169.30, 157.12, 156.27, 154.56, 149.50, 149.12, 142.29, 136.66, 135.87, 133.38, 129.89, 129.16, 128.70, 128.09, 127.63, 127.38, 121.98, 121.27, 118.92, 118.24, 116.81, 112.79, 112.35, 111.71, 110.16, 66.13, 58.87, 55.12, 55.05,

42.26, 25.68.

Tert-butyl(2-(2-(4-bromo-3-hydroxyphenyl)-5-(methylcarbamoyl)-1H-benzo[d]imidazol-1-yl)-2-(3,4-dimethoxyphenyl)ethyl)carbamate (**5b**). **5b** was synthesis the same as **P21**. Light yellow solid, yield 42%. ^{1}H NMR (400 MHz, CD_3OD) δ 8.18 (d, J = 12.4 Hz, 1H), 7.70 (d, J = 8.6 Hz, 1H), 7.60 (d, J = 8.1 Hz, 1H), 7.37 (d, J = 8.6 Hz, 1H), 7.16 (d, J = 13.0 Hz, 1H), 6.95 (dd, J = 15.4, 7.8 Hz, 2H), 6.84 (d, J = 8.1 Hz, 2H), 5.91 (d, J = 5.4 Hz, 1H), 4.06 (dd, J = 14.3, 4.0 Hz, 1H), 3.92 (d, J = 10.4 Hz, 1H), 3.82 (s, 3H), 3.72 (s, 3H), 2.94 (s, 3H), 1.16 (d, J = 84.3 Hz, 9H).

2-(4-bromo-3-methoxyphenyl)-1-(2-(2-(2-(tert-butyl)phenoxy)acetamido)-1-(3,4-dimethoxyphenyl)ethyl)-N-methyl-1H-benzo[d]imidazole-5-carboxamide (**P80**). **5b** (480 mg, 0.76 mmol) was solved in 5 mL 1,4-dioxane, and the mixture was added 2 mL HCl (2 N in 1,4-dioxane). The reaction was stirred at rt for 4 h and then concentrated. The residue was redissolved in N,N-dimethylformamide (6 mL), and was added 2-(2-(*tert*-butyl)phenoxy)acetic acid (79.9 mg, 0.38 mmol), 1-[bis(dimethylamino)methylene]-1*H*-1,2,3-triazolo[4,5-*b*]pyridinium 3-oxide hexafluorophosphate (HATU) (218.8 mg, 0.57 mmol) and DIPEA (0.204 mL, 1.15 mmol) at 0 °C. The reaction was stirred at rt for 2 hours. After finished, the reaction was concentrated. The residue was purified with flash chromatography to afford **P80** (100.2 mg, 36% yield) as a white solid. ^{1}H NMR (500 MHz, CD_3OD) δ 8.21 (s, 1H), 7.75 (d, J = 8.5 Hz, 1H), 7.59 (d, J = 8.0 Hz, 1H), 7.52 (d, J = 8.5 Hz, 1H), 7.21 (d, J = 7.6 Hz, 1H), 7.07 (dd, J = 15.2, 7.3 Hz, 2H), 6.95–6.86 (m, 3H), 6.84 (s, 2H), 6.57 (d, J = 8.1 Hz, 1H), 6.06 (dd, J = 8.8, 5.3 Hz, 1H), 4.38 (dd, J = 12.5, 6.2 Hz, 1H), 4.33 (d, J = 4.1 Hz, 2H), 4.22 (dd, J = 13.7, 10.2 Hz, 1H), 3.81 (s, 3H), 3.70 (s, 3H), 2.95 (s, 3H), 1.19 (s, 9H). ^{13}C NMR (126 MHz, CD_3OD) δ 170.33, 169.15, 156.62, 155.90, 154.69, 149.58, 149.25, 142.24, 138.25, 135.95, 133.53, 129.76, 129.31, 128.66, 127.02, 126.42, 122.28, 121.58, 121.10, 118.88, 118.38, 116.59, 113.20, 112.71, 112.61, 111.80, 110.14, 67.55, 58.75, 55.11, 55.07, 40.79, 34.03, 29.07, 25.65.

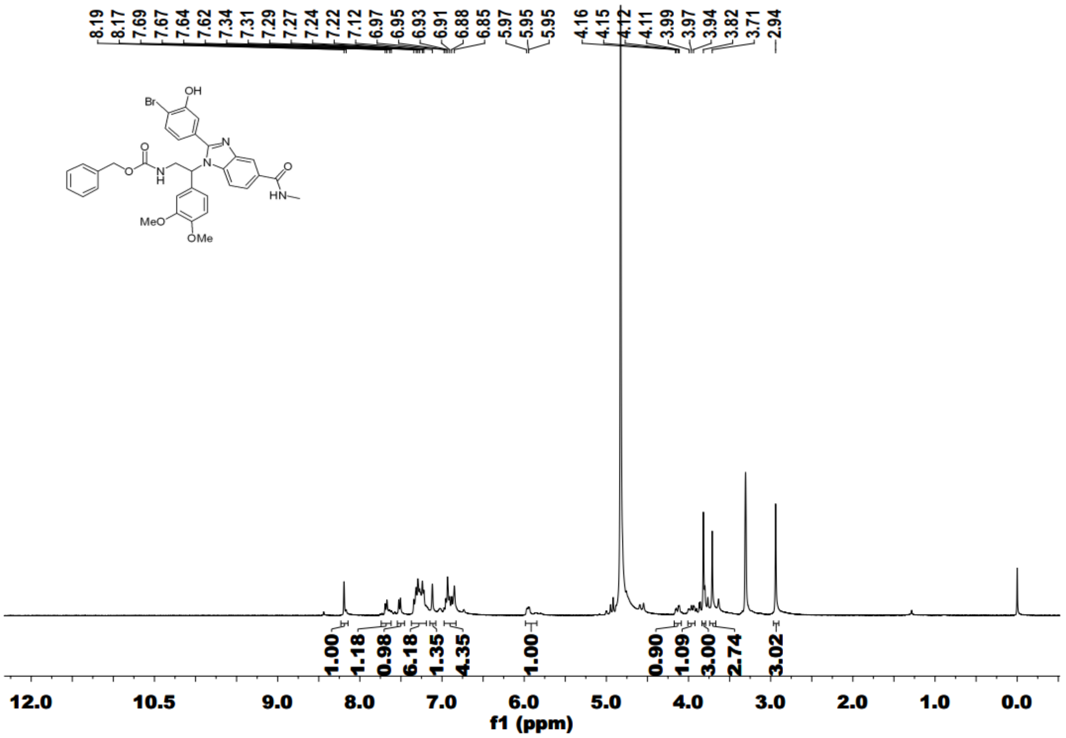

^{1}H-NMR spectra of **P21**

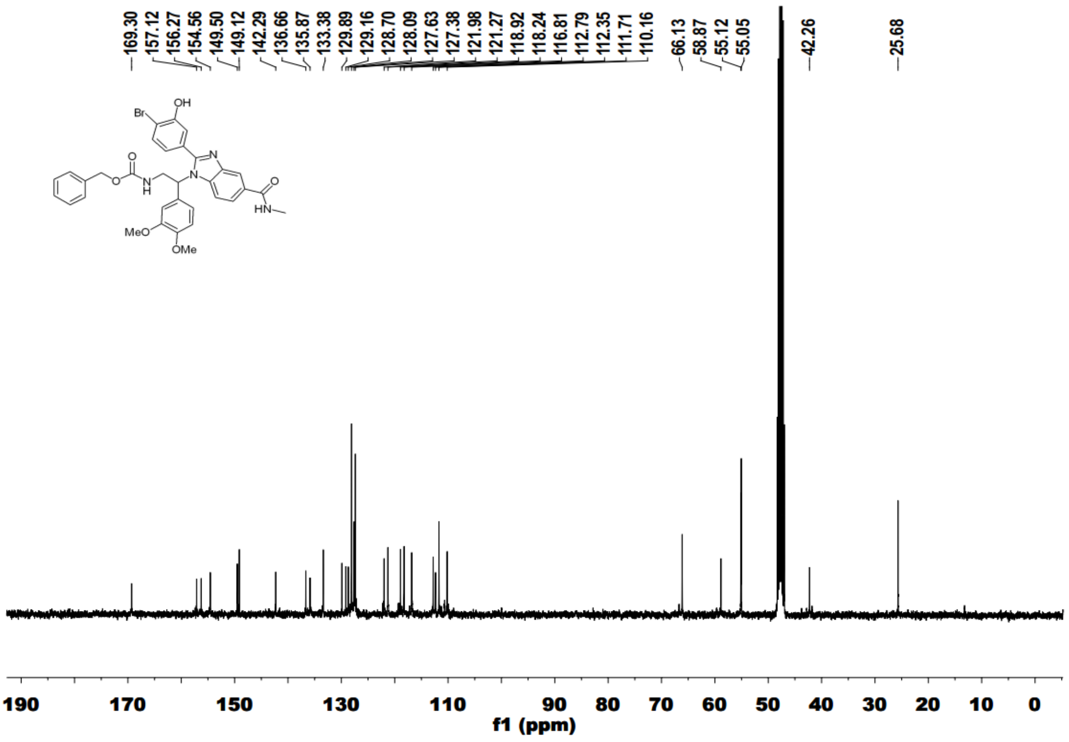

^{13}C-NMR spectra of **P21**

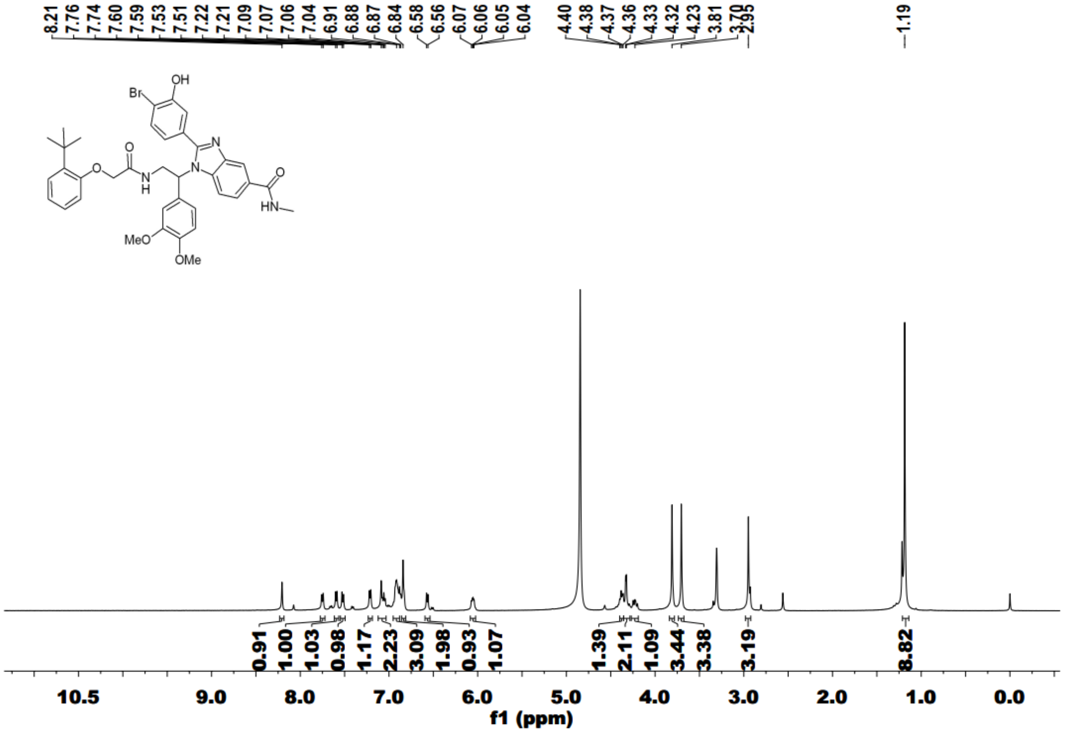

^{1}H-NMR spectra of **P80**

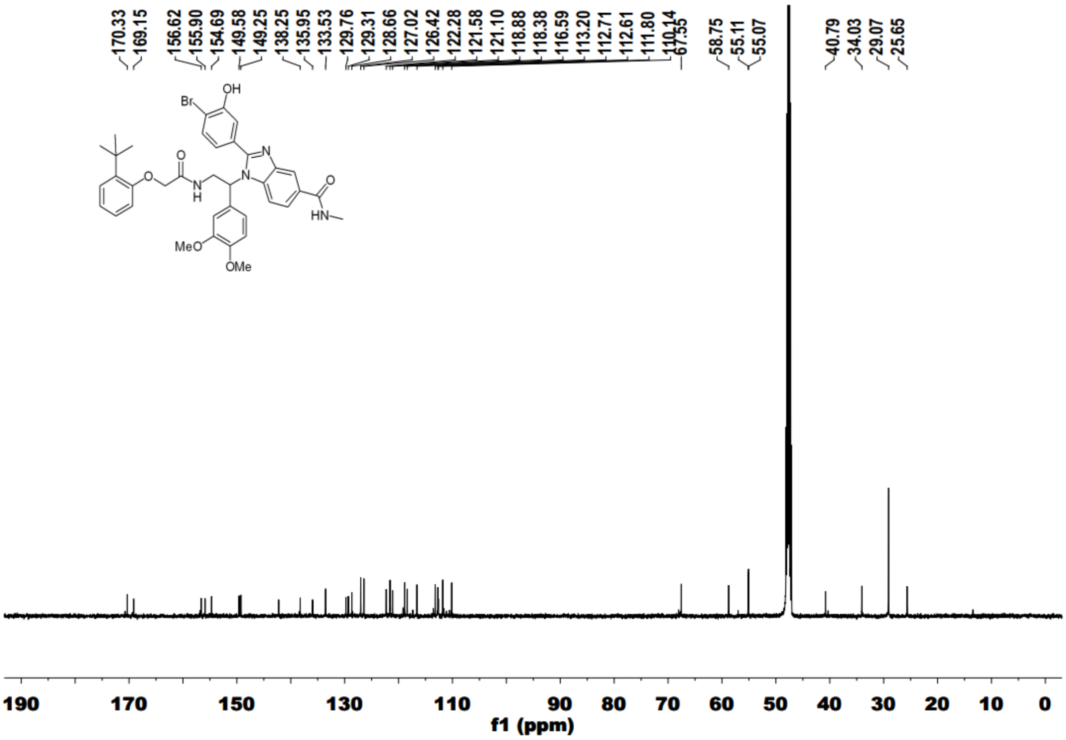

^{13}C-NMR spectra of **P80**

Supplementary References

64. Katoh, K., Misawa, K., Kuma, K. and Miyata, T. (2002) MAFFT: a novel method for rapid multiple sequence alignment based on fast Fourier transform. *Nucleic Acids Res*, **30**, 3059-3066.
65. Lawson, E.C., Luci, D.K., Ghosh, S., Kinney, W.A., Reynolds, C.H., Qi, J., Smith, C.E., Wang, Y., Minor, L.K., Haertlein, B.J. *et al.* (2009) Nonpeptide urotensin-II receptor antagonists: a new ligand class based on piperazino-phthalimide and piperazino-isoindolinone subunits. *J Med Chem*, **52**, 7432-7445.
